# Enhancement of isoflavone aglycones, GABA, and mineral bioavailability in *Apios americana* Medikus by co-fermentation with *Lactiplantibacillus plantarum* LAB02 and *Levilactobacillus brevis* BMK484

**DOI:** 10.1016/j.fochx.2026.103745

**Published:** 2026-03-10

**Authors:** Hee Yul Lee, Hyo Seon Kim, Ga Young Lee, Young Hye Seo, Du Yong Cho, Jong Bin Jeong, Mu Yeun Jang, Da Hyun Kim, Do Yun Bang, Hye Rim Kim, Ye Rim Jeong, Jun Lee, Kye Man Cho

**Affiliations:** a Department of GreenBio Science (BK21 Four) and Agri-Food Bio Convergence Institute, Gyeongsang National University, Naedong-ro 139-8, Jinju 52849, Republic of Korea; b Gyeongnam Anti-Aging Research Institute, 69 Chinhwangyeng-ro 2605beon-gil, Sancheong 52215, Republic of Korea; c Herbal Medicine Resources Research Center, Korea Institute of Oriental Medicine (KIOM), Geonjae-ro 111, Naju, 58245, Republic of Korea; d Division of Food Science and Technology, Gyeongsang National University, Naedong-ro 139-8, Jinju 52849, Republic of Korea

**Keywords:** *Apios americana* Medikus, Lactic acid bacteria, Isoflavone, Amino acid, Fatty acid, Antioxidant

## Abstract

This study evaluated metabolic and functional changes in *Apios americana* Medikus (AAM) during lactic acid bacteria fermentation using *Lactiplantibacillus plantarum* LAB02 and *Levilactobacillus brevis* BMK484 at different strain ratios and fermentation times. A 1:1 mixed culture fermented for 36 h was optimal. Isoflavone glycosides decreased (1.837 → 0.006 mg/g), while aglycones increased (0.097 → 0.604 mg/g), including genistein (0.194 mg/g) and 2′-hydroxygenistein (0.184 mg/g). Fermentation increased γ-aminobutyric acid (52.6 mg/100 g), aspartic acid, and extractable minerals (P, K, Ca, Mg) under the applied extraction conditions, with key unsaturated fatty acids retained. Antioxidant activity improved, with DPPH and ABTS IC₅₀ values reduced by 26.7% and 21.5%, respectively. PCA and heatmap clustering confirmed distinct shifts among dried, sterilized, and fermented AAM, supporting its use as a functional food ingredient.

## Highlights

• Aglycone contents of AAM were maximized by fermentation at 30 °C for 36 h using a 1:1 mixed culture of LAB02 and BMK484.
• FeAAM simultaneously enhanced GABA (52.6 mg/100 g) and isoflavone aglycone (0.996 mg/g) contents.
• Isoflavones were stepwise converted from diglycosides to aglycones from DrAAM–StAAM–FeAAM.
• TPC, antioxidant activities, and digestive enzyme inhibitory activities increased progressively from DrAAM to StAAM to FeAAM.

## 1 Introduction

*Apios americana* Medikus (AAM), also known as American groundnut, is a tuberous leguminous plant traditionally consumed in North America and East Asia. AAM tubers contain substantial amounts of starch, proteins, minerals, and distinctive isoflavones (e.g., genistein-7-O-gentiobioside [GGB]), which have been associated with various biological activities, including antioxidant, anti-inflammatory, and neuroprotective effects (Takashima et al., 2013; Zhou et al., 2023).

Recent studies have extensively characterized the isoflavone profile of AAM tubers, and specific compounds, including 2′-hydroxygenistein (HGE) derivatives, were shown to exert cytoprotective effects via Nrf2/HO-1 signaling pathways (Zhou et al., 2023). In addition, fungal fermentation using several species has been reported to convert glycosidic isoflavones into more bioavailable aglycone forms and to enhance metabolite levels and antioxidant activity in AAM (Lee, Kim, et al., 2024). However, fungal solid-state fermentation is sensitive to temperature, oxygen, and moisture, making process control difficult; it also requires longer fermentation times and carries a higher risk of contamination (Pandey, 2003). By contrast, lactic acid bacteria (LAB) fermentation is generally faster, easier to control, and more suitable for standardized food production.

LAB fermentation has gained increasing attention as a safe and food-compatible bioprocessing strategy. LAB are generally recognized as safe and are widely used in functional food development because of their ability to improve flavor, shelf life, and nutritional value. Notably, certain LAB strains, including *Lactiplantibacillus plantarum* and *Levilactobacillus brevis*, exhibit strong β-glucosidase activity, enabling the hydrolysis of glycosidic isoflavones into more bioavailable aglycones (Cho et al., 2022). Moreover, these strains can produce γ-aminobutyric acid (GABA), a bioactive compound linked to hypotensive and neuroprotective effects (Hwang et al., 2021).

To our knowledge, this is the first study to apply LAB-based co-fermentation to AAM. Using a mixed culture of *L. plantarum* and *L. brevis*, we demonstrate the simultaneous enhancement of isoflavone aglycones and GABA in a starch-rich tuber matrix. Notably, this outcome contrasts with fungal AAM fermentation, which has been reported to show strain-dependent metabolite shifts, including an opposite glutamic acid/GABA trend (i.e., increased glutamic acid with decreased GABA) despite high aglycone conversion. Therefore, the present study investigated LAB-driven bioconversion and functional changes in AAM by analyzing viable LAB counts, isoflavones, GABA production, and antioxidant capacity (2,2-diphenyl-1-picrylhydrazyl [DPPH] and 2,2′-azino-bis(3-ethylbenzthiazoline-6-sulfonic acid) [ABTS] assays). Multivariate statistical analyses, including principal component analysis (PCA) and hierarchical clustering, were additionally employed to evaluate metabolic transitions during fermentation.

## 2 Materials and methods

### 2.1 Materials, strains, reagents and instruments

#### 2.1.1 Plants

AAM tubers (cultivated in Jiphyeon-myeon, Jinju-si, Gyeongsangnam-do, Republic of Korea) were purchased for use in this study. The tubers were thoroughly rinsed with water before processing to remove surface soil and impurities. Fresh AAM tubers were thoroughly washed and cut into approximately 1 cm cubes (width × length × height), including the peel. Then, the chopped tubers were dried using a hot-air dryer (WOF-W155, DAIHAN Scientific, Korea) at 55 °C for 48 h. After drying, the samples were stored at −40 °C until further use. Prior to the experiment, the dried samples were pulverized into a fine powder using a laboratory grinder and passed through a 100-mesh sieve to ensure uniform particle size.

#### 2.1.2 Strains and growth media used for fermentation

Four LAB strains were used for AAM fermentation, including L. *plantarum* LAB02 (LAB02), *L. brevis* WCP02 (WCP02), *L. brevis* BMK184 (BMK184), and *L.* *brevis* BMK484 (BMK484). All strains were previously isolated from fermented foods and kimchi and maintained in the laboratory. Each LAB strain was cultured in Lactobacilli MRS Broth (BD, New Jersey, NJ, USA) at 30 °C for 48 h using a shaking incubator (ThermoStable IR-250, DAIHAN Scientific, Korea) and subsequently used for AAM fermentation.

#### 2.1.3 Reagents and instruments

All reagents used in this study were of analytical or high-performance liquid chromatography (HPLC) grade. 5-Sulfosalicylic acid dihydrate was purchased from Sigma-Aldrich (St. Louis, MO, USA) and used for dissolving the concentrated solution during sample pretreatment in free amino acid (FAA) analysis. Other reagents used for total phenolic content (TPC), total flavonoid content (TFC), and antioxidant activity assays, including 2 N Folin–Ciocalteu's phenol reagent, diethylene glycol, DPPH, and ABTS, were also obtained from Sigma-Aldrich. Solvents that were used in sample extraction and instrumental analyses, including acetic acid, distilled water, methanol, and acetonitrile, were purchased from J.T. Baker (Phillipsburg, NJ, USA). Additional reagents were procured as needed for specific analyses.

The fatty acid composition was determined using gas chromatography (GC, Nexis GC-2030, Shimadzu Corp., Kyoto, Japan). The contents of FAAs were analyzed with an automatic amino acid analyzer (L-8900, Hitachi High-Technologies Corp., Tokyo, Japan). The content of minerals was measured using inductively coupled plasma mass spectrometry (NexION 350, PerkinElmer Inc., Waltham, MA, USA). Isoflavone compounds were quantified using ultra-performance liquid chromatography (UPLC, Acquity UPLC H-Class Plus System, Waters, Milford, MA, USA). The OD value of start culture, TPC, TFC, antioxidant activities, and digestive enzyme inhibitory activities were spectrophotometrically analyzed using a UV-1800 spectrophotometer (Shimadzu Corp., Kyoto, Japan).

### 2.2 Optimization of fermentation conditions using various LAB

#### 2.2.1 Fermentation

AAM fermentation conditions were optimized in three steps: (i) single strain/strain-combination screening, (ii) inoculum-ratio optimization, and (iii) time-course fermentation. Powdered dried AAM (DrAAM, 100 g) and water (100 mL) were sterilized separately (121 °C, 15 min), aseptically mixed, and fermented at 30 °C. For all experiments, the total inoculum was set at 5% (*v*/*w*). Starter cultures were adjusted to a final concentration of 10^9^ CFU/mL based on spectrophotometric calibration at OD 600 nm, followed by resuspension in sterile phosphate-buffered saline (PBS). In Step 1, individual strains (LAB02, WCP02, BMK184, and BMK484) and co-cultures (LAB02 + WCP02, LAB02 + BMK184, LAB02 + BMK484) were fermented for 72 h, with not fermentation AAM (CTL, control). LAB02 and BMK484 were selected for subsequent ratio/time optimization based on an initial screening of single starters and LAB02-based co-cultures, which evaluated isoflavone bioconversion outcomes. In Step 2, LAB02 and BMK484 were co-inoculated at ratios of 1:1, 1:2, 1:3, 2:1, and 3:1 and fermented for 72 h. In Step 3, samples were collected at 0, 12, 24, 36, 48, 60, and 72 h under the selected condition. Fermented samples were dried (55 °C, 48 h), pulverized, and stored at −40 °C until analysis.

#### 2.2.2 16S rRNA of LAB02 strains

The LAB02 strain was identified by amplifying the 16S rRNA gene using the universal primer pair 877F/878R, which is commonly used for *Lactobacillus* species identification (Hou & Kim, 2020). The PCR products were sequenced and compared to reference sequences in the NCBI database using BLAST. Phylogenetic relationships were inferred using DNAMAN software (v. 4.11) with the neighbor-joining method and 1000 bootstrap replicates (Huang et al., 2025).

#### 2.2.3 Analysis of isoflavones

Isoflavones were extracted and quantified in accordance with the method of Lee, Kim et al. (2024). Briefly, 0.2 g of powdered sample was mixed with 10 mL of 30% ethanol and subjected to ultrasonic extraction for 1 h at room temperature. Then, the extract was filtered through a 0.45-μm membrane filter prior to chromatographic analysis. UPLC was performed using a Waters Acquity UPLC system equipped with a BEH Phenyl column (2.1 mm × 150 mm, 1.7 μm; Waters, Milford, MA, USA). The column was maintained at 35 °C, and 2 μL of the sample was injected. The mobile phase comprised solvent A (ultrapure water with 0.05% formic acid) and solvent B (acetonitrile). The following gradient program was applied: initial 5% B, ramped to 30% B at 18 min, and then to 100% B at 20 min. The flow rate was 0.3 mL/min, and isoflavones were detected at 254 nm. Quantification was performed using external standard calibration. Standard curves were constructed from six concentrations (0.1–1.0 mg/mL) of each isoflavone standard (stock solution: 1.0 mg/mL) and fitted by linear regression. All calibration curves showed excellent linearity (R^2^ = 0.999–1.000; *n* = 5). In addition, the retention times of sample peaks were matched to those of authentic standards for identification. The concentrations were expressed as mg/g. Supplementary Table S1 presents the details of the calibration parameters. Typical UPLC chromatograms of 13 isoflavone derivatives and chemical structures was according to Supplementary Fig. S1.

### 2.3 Characteristics of AAM according to the food processing stages

#### 2.3.1 AAM processing conditions

AAM processing proceeded with drying, sterilization, and fermentation (Supplementary Fig. S2). First, DrAAM was dried for 48 h at 55 °C and ground to a 100-mesh size. Second, DrAAM and distilled water (one-fold of the DrAAM weight, *v*/*w*) were sterilized at 121 °C for 15 min and mixed. The sample was named StAAM. Finally, the mixture was fermented with starter cultures of LAB02 (2.5% *v/w*) and BMK484 (2.5% *v/w*) at 30 °C for 72 h. Then, StAAM and FeAAM were dried for 48 h at 55 °C and ground to a 100-mesh size. DrAAM, StAAM, and FeAAM were stored at −40 °C until use in the experiments.

#### 2.3.2 Physicochemical properties and viable cell numbers

The pH was measured using a pH meter (Orion Star A211, Thermo Fisher Scientific Inc., Waltham, MA, USA). The total acidity was determined by titrating 1 g of each sample with 0.1 N NaOH to a final pH of 8.2 ± 0.1. The amount of NaOH used was converted to lactic acid content and expressed as % lactic acid. The content of soluble solids was measured using a digital refractometer (PR-101α, ATAGO Co., Ltd., Tokyo, Japan) and expressed as °Brix (%) (Lee, Kim, et al., 2024).

The plate count method was used to determine viable cell counts in fermented samples. Briefly, 1.0 g of each sample was aseptically homogenized with 9.0 mL of sterile saline (0.85% NaCl) to prepare a 10^−1^ suspension, followed by serial 10-fold dilutions prepared as needed (typically 10^−1^–10^−8^) to obtain countable plates. An aliquot of each appropriate dilution (0.1 mL) was spread-plated on MRS agar and incubated at 30 °C for 48 h under anaerobic conditions. Plates containing 30–300 colonies were used for enumeration. Viable cell counts were calculated as eq. (1):

(1) $$\mathrm{CFU}/g=\left(C\times \mathrm{DF}\right)/\left(V\times m\right)$$

where C is the colony count, DF is the dilution factor, V is the plated volume (mL), and m is the sample mass (g). Results were expressed as CFU/g.

Moisture content of FeAAM was determined gravimetrically using mass measurements taken before fermentation (*W₀*), immediately after fermentation (*W*_*f*_), and after drying to constant mass (*W*_*d*_; 55 °C, 48 h). The moisture content was calculated by using eq. (2) and expressed as percentage.

(2) $$\text{Moisture content}\;\left(\%,\mathrm{wet}\;\text{basis}\right)=\left(W-W_{d}\right)/W\times 100$$

where *W* represents *W₀* or *W*_*f*_. Moisture loss during fermentation was evaluated as (moisture content % of *W₀* − moisture content % of *W*_*f*_).

#### 2.3.3 Analysis of fatty acids

After the methyl esterification of lipid extracts, the fatty acid composition was analyzed in accordance with the method of Lee, Lee, et al. (2024) with modifications. For derivatization, 25 mg of sample powder was treated with 0.5 mL of 0.5 N sodium hydroxide in methanol and 0.5 mL of triundecanoin (C11:0, 2 mg/mL) as the internal standard. The mixture was then vortexed and incubated at 100 °C for 5 min to promote hydrolysis. Subsequently, 2 mL of 14% boron trifluoride in methanol (Supelco, Bellefonte, PA, USA) was introduced, and the mixture was heated at the same temperature for 30 min to induce esterification. After cooling, the mixture was added with 1 mL of isooctane, followed by 5 mL of saturated sodium chloride solution. The upper organic layer was separated, dried over anhydrous sodium sulfate, and filtered using a 0.45-μm membrane filter (Dismic-25CS, Toyoroshikaisha, Tokyo, Japan). GC analysis was conducted using a flame ionization detector and an SP-2560 capillary column (100 m × 0.25 mm, 0.2 μm film, Supelco, St. Louis, MO, USA). The injector was operated at 225 °C in the split mode (200:1), with helium as the carrier gas at a flow rate of 0.75 mL/min. The column oven was programmed from 100 °C (4-min hold) to 240 °C at 3 °C/min, followed by a 15 min hold. Detection was performed at 285 °C. Fatty acids were identified by comparing the retention times with a commercial standard mix (CRM47885, Supelco 37 Component FAME Mix, Sigma Aldrich, St. Louis, MO, USA) and expressed as mg/100 g.

#### 2.3.4 Analysis of free amino acids

FAA quantification was performed using a modified hydrolysis-based extraction (Lee, Lee, et al., 2024). Approximately 100 mg of sample powder was suspended in 5 mL of distilled water and incubated at 60 °C for 1 h. Thereafter, the hydrolyzed mixture was treated with 1 mL of 10% (*w*/*v*) 5-sulfosalicylic acid dihydrate and allowed to stand at 4 °C for 2 h to precipitate the proteins. After centrifugation (15,000 rpm, 3 min), the clear supernatant was passed through a syringe filter and subsequently concentrated under vacuum at 55 °C using a rotary evaporator. The residue was reconstituted in 2 mL of lithium citrate buffer (pH 2.2) and then filtered through a 0.45-μm membrane. Amino acid analysis was performed with an automated amino acid analyzer, using a certified standard mixture (Type H, Wako Pure Chemical Industries Ltd., Osaka, Japan) for calibration. The results were reported as mg/100 g dry weight.

#### 2.3.5 Analysis of minerals

For mineral analysis, sample pretreatment was performed as follows: a portion of each sample powder (0.5 g) was mixed with 10 mL of nitric acid solution and subjected to microwave-assisted digestion. After complete decomposition, the mixture was diluted with sterilized distilled water to a final volume of 50 mL to prepare the test solution. For sodium analysis, a separate dry ashing method was used. Approximately 0.5 g of the sample was incinerated in a muffle furnace at 550–600 °C until white or off-white ash was obtained. The ash was cooled and then dissolved in 3% nitric acid. The final volume was adjusted to 30 mL before analysis. Standard calibration curves were generated using certified standards for each mineral, and the concentrations were expressed in mg/100 g of sample (Lee, Lee, et al., 2024).

#### 2.3.6 Extract preparation

One gram of each sample powder was mixed with 20 mL of 50% methanol and vortexed. Then, the mixture was extracted by stirring at room temperature for 12 h. After centrifugation at 4000 rpm for 10 min, the supernatant was filtered through a 0.45-μm membrane filter and used for the analyses of isoflavones, TPC, TFC, and radical scavenging activity.

#### 2.3.7 Analysis of TPC and TFC

The TPC was assessed using a modified Folin–Denis assay based on the method of Lee et al. (2022). Gallic acid was used to construct a calibration curve (0.02–0.10 mg/mL). For the analysis, 0.5 mL of each sample or standard solution was combined with 0.25 mL of 2 N Folin–Ciocalteu reagent and incubated for 3 min at room temperature. Subsequently, the sample was added with 0.5 mL of 25% sodium carbonate solution, and the reaction mixture was incubated at 30 °C for 1 h. After centrifugation at 13,000 rpm for 1 min, the absorbance was recorded at 750 nm using an ultraviolet–visible (UV–Vis) spectrophotometer (UV-1800, Shimadzu, Kyoto, Japan). The TPC values were calculated using the gallic acid calibration curve and expressed as mg gallic acid equivalents per gram of sample.

The TFC was determined in accordance with the method of Lee et al. (2022) with slight modifications. Rutin was used as the reference compound, and standard solutions were prepared at concentrations of 0.02–0.10 mg/mL. For each assay, 0.5 mL of the sample or standard was mixed with 1 mL of diethylene glycol and briefly vortexed. Then, 0.01 mL of 1 N NaOH was added, and the mixture was incubated at 37 °C for 1 h. The absorbance was measured at 420 nm using a UV–Vis spectrophotometer. The results were calculated based on the standard curve of rutin and presented as mg rutin equivalents per gram of sample.

#### 2.3.8 Analysis of antioxidant activities

The radical scavenging activities of fermented AAM was evaluated in accordance with the method of Cho et al. (2025), The radical scavenging activities of fermented AAM was evaluated in accordance with the method of Cho et al. (2025), and the results were expressed as half-maximal inhibitory concentration (IC₅₀) values in accordance with the procedure of Adinortey et al. (2020). The IC₅₀ values for DPPH and ABTS radical scavenging activities were determined by measuring the absorbance of various sample extract concentrations and the negative control.

For the DPPH radical scavenging assay, 0.2 mL of each sample was mixed with 0.8 mL of 1.5 × 10^−4^ M DPPH solution. Then, the mixture was incubated in the dark for 30 min, and the absorbance was measured at 525 nm using a UV–Vis spectrophotometer.

For the ABTS radical scavenging assay, 7 mM ABTS solution and 2.45 mM potassium persulfate were mixed at a 1:3 ratio and incubated in the dark for 14 ± 2 h to generate ABTS^+^ radicals. Then, the resulting solution was diluted with methanol to obtain an absorbance of 0.70 ± 0.03 at 732 nm. Each sample extract (0.1 mL) was mixed with 0.9 mL of the diluted ABTS^+^ solution and reacted in the dark for 3 min. The absorbance was measured at 732 nm using a spectrophotometer. The negative control was prepared using 0.1 mL of distilled water instead of the sample. The DPPH and ABTS radical scavenging activities were calculated as a percentage (%) using the following eq. (3), and the IC₅₀ values were determined accordingly.

(3) $$\text{Radical scavenging acitivity}\;\left(\%\right)=\left[1-\left(A_{\mathrm{sample}}/A_{\mathrm{control}}\right)\right]\times 100$$

Where *A*_*sample*_ and *A*_*control*_ are the absorbance of the sample and negative control, respectively.

The ferric reducing antioxidant power (FRAP) of fermented AAM was determined according to Lee, Cho et al. (2023). The FRAP working reagent was freshly prepared by mixing acetate buffer (300 mM, pH 3.6), TPTZ (10 mM), and FeCl₃ (20 mM) at a ratio of 10:1:1 (*v/v/v*), followed by pre-incubation at 37 °C for 15 min. The sample (0.05 mL) was mixed with FRAP reagent (0.95 mL) and incubated at 37 °C for 15 min, and the absorbance was measured at 590 nm. The calibration curve was constructed using FeSO₄·7H₂O, and FRAP values were expressed as FeSO₄·7H₂O equivalents (mg/g sample) using the following eq. (4):

(4) $$\text{FRAP}\;\left(\mathrm{mg}\;\text{FeSO}₄\cdot 7H₂O/g\right)=\frac{\left(y-0.0972\right)\times V_{\mathrm{ext}}\times \mathrm{DF}}{0.004\times W\times 1000}$$

where *y* is the absorbance at 590 nm, *V*_*ext*_ is the extraction volume, *DF* is the dilution factor, *W* is the sample weight, and 1000 is factor relating μg to mg.

#### 2.3.9 Analysis of digestive enzyme inhibitory activities

To assess digestive enzyme inhibitory activities (α-glucosidase and pancreatic lipase), the assays were conducted following the previously reported procedure of Lee, Lee, et al. (2024). The reactions were stopped by adding 750 μL of 100 mM Na₂CO₃, and absorbance was recorded at 420 nm. For the negative control, the sample solvent was substituted for the sample; absorbance values from both the test and negative control were used to calculate inhibition (%) according to eq. (5).

(5) $$\text{Digestive enzyme inhibitory activity}\;\left(\%\right)=\left[1-\left(A_{\mathrm{sample}}/A_{\mathrm{control}}\right)\right]\times 100$$

where *A*_*sample*_ is the absorbance of the sample and *A*_*control*_ is the absorbance of the control reaction.

### 2.4 Statistical analysis and data processing

The experimental results are presented as mean ± standard deviation from three independent biological replicates (*n* = 3). Statistical analyses were performed using SPSS (version 12.0). Differences among groups were evaluated by one-way ANOVA followed by Duncan's multiple-range test (*p* < 0.05). Multivariate analyses were conducted using MetaboAnalyst 6.0. Data were auto-scaled prior to analysis. Principal component analysis (PCA) was performed and visualized with 95% confidence intervals. Heat maps were generated using Euclidean distance and Ward's linkage visualization (Chong et al., 2019).

## 3 Results and discussion

### 3.1 Comparison of isoflavones in AAM according to fermentation conditions

Based on previous studies demonstrating their high GABA-producing capacity and physiological activity, the strains (WCP02, BMK184 and BMK484) were selected to determine the optimal fermentation conditions for AAM (Lee et al., 2021). In addition, the LAB02, strain was included for AAM fermentation. The nucleotide sequence of the LAB02 strain was identified using 16S rRNA gene sequencing (Supplementary Fig. S3). Phylogenetic analysis based on 16S rRNA sequence alignment confirmed its taxonomic position, indicating that LAB02 was most closely related to *L.* *plantarum* GV64 (Supplementary Fig. S4).

**Fig. 1:**
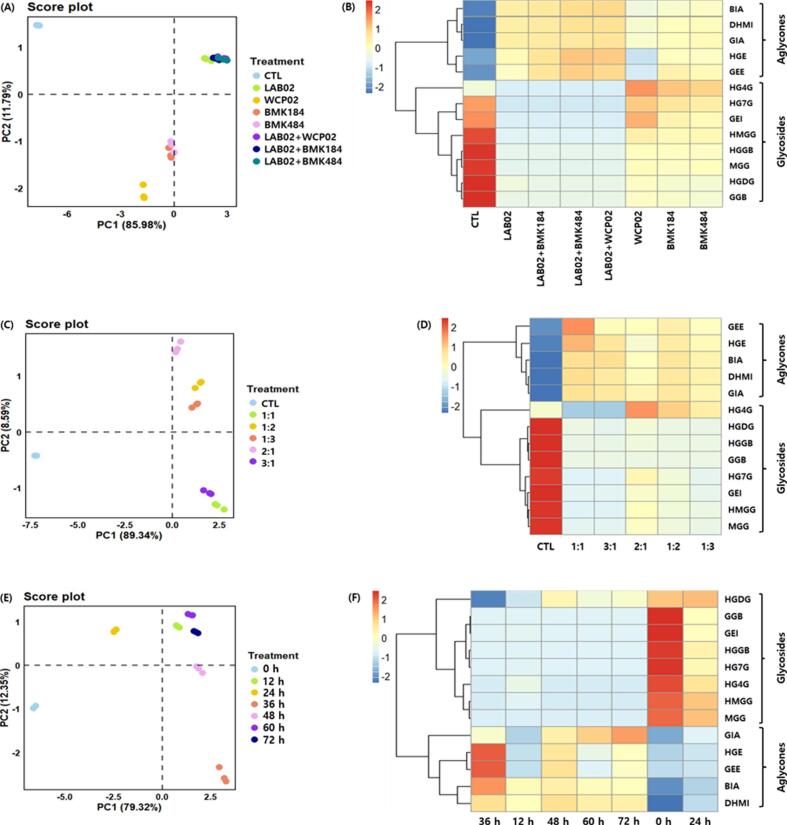
Comparison of isoflavones in *Apios americana* Medikus (AAM) according to fermentation with the single and mixed starters of LAB, strain ratio of LAB, and fermentation times. (A) score plot of PCA and (B) correlation heatmap analysis of the single and mixed starters of LAB, (C) score plot of PCA and (D) correlation heatmap analysis of strain ratio of LAB, and (E) score plot of PCA of and (F) correlation heatmap analysis of fermentation times. Fermentation conditions: (A) and (B), the AAM was fermented for 72 h at 30 °C using LAB (such as CTL, control; *L. plantarum* LAB02; *L. brevis* WCP02; *L. brevis* BMK184; *L. plantarum* LAB02 + *L. brevis* WCP02; *L. plantarum* LAB02 + *L. brevis* BMK184; *andL.plantarum* LAB02 + *L. brevis* BMK484); (C) and (D), the AAM was fermented for 72 h at 30 °C using a mixture of various ratios of *L.plantarum* LAB02 and *L.brevis* BMK 484 (such as LAB02:BMK484 = 1:1; 1:2; 1:3; 2:1; and 3:1), and (E) and (F), the AAM was fermented at 30 °C using a 1:1 mixture of *L.plantarum* LAB02 and *L. brevis* BMK184 during fermentation (such as 0; 12; 24; 36; 48; 60; and 72 h). Isoflavones: HGDG, 2′-hydroxygenistein-4′,7-*O*-diglucoside; HMGG, 2′-hydroxy, 5-methoxygenistein-7-*O*-glucoside; HGGB, 2′-hydroxygenistein-7-*O*-gentibioside; MGG, 5-methoxygenistein-7-*O*-gucoside; HG7G, 2′-hydroxygenistein-7-*O*-glucoside; GGB, genistein-7-*O*-gentibioside; HG4G, 2′-hydroxygenistein-4′-*O*-glucoside; GEI, genistin; BIA, barpisoflavone A; DHMI, 4′,7-dihydroxy-5-methoxyisoflavone; GIA, gerontoisoflavone; HGE, 2′-hydroxygenistein; and GEE, genistein. The values of various conditions were normalized and clustered in the heatmap. The color displays the intensity of the normalized mean values of different parameters. Statistical significance was considered at *p* < 0.05.

Isoflavone profiles (13 derivatives) in AAM fermented with single or mixed starters, different inoculum ratios, and varying fermentation times are summarized in Fig. 1 and Supplementary Tables S2–S4. In the single-starter fermentations (Table S2), total glycosides decreased markedly from 3.085 mg/g in the non-fermented control (CTL) to 0.015 (LAB02), 0.815 (WCP02), 0.693 (BMK184), and 0.626 mg/g (BMK484), and glycosides were not detected in the LAB02-based co-fermentations (LAB02 + WCP02, LAB02 + BMK184, LAB02 + BMK484). In contrast, total aglycones increased from 0.096 mg/g (CTL) to 1.026, 0.639, 1.030, 0.992, 1.327, 1.238, and 1.348 mg/g across fermented groups. PCA (Fig. 1A) showed clear separation between CTL and all fermented samples (PC1, 85.95%; PC2, 11.79%), indicating substantial fermentation-driven remodeling of isoflavone composition. LAB02 alone clustered closely with LAB02-based co-fermentations, and the heatmap (Fig. 1B) consistently showed higher aglycone abundance in these groups relative to CTL.

Based on the screening results (Table S2), LAB02 + BMK484 was selected for further optimization due to its favorable aglycone-dominant profile. Isoflavone composition under different LAB02:BMK484 ratios is presented in Fig. 1C–D and Supplementary Table S3. Total isoflavones decreased after fermentation compared with the non-fermented control (2.828 mg/g). Across all ratios, glycosides decreased by >84.8% relative to CTL (2.606 mg/g), whereas aglycones increased significantly, ranging from a 5.4-fold increase (1.202 mg/g at 1:2) to a 7.0-fold increase (1.542 mg/g at 1:1) compared with CTL (0.222 mg/g). PCA separated CTL from fermented samples (PC1, 89.34%; PC2, 8.59%) and further discriminated groups by inoculum ratio, indicating ratio-dependent metabolic differences. Heatmap clustering showed distinct glycoside/aglycone patterns between CTL and fermented samples, with the 1:1 and 3:1 ratios exhibiting similar profiles. Given its highest aglycone conversion, the 1:1 ratio was selected for time-course experiments.

Fermentation time strongly influenced isoflavone remodeling (Supplementary Table S4). Total glycosides decreased from 1.837 mg/g at 0 h to 0.006 mg/g at 36 h, followed by a slight increase at later time points. Conversely, total aglycones increased from 0.097 mg/g at 0 h to 0.604 mg/g at 36 h. Notably, HGE (0.184 mg/g) and genistein (GEE, 0.194 mg/g) reached their highest levels at 36 h, indicating maximal conversion at this time point. PCA showed clear time-dependent separation (PC1, 79.32%; PC2, 12.35%), with early-stage samples (0−12 h) clustering apart from later-stage samples (>36 h), consistent with the heatmap patterns (Fig. 1E–F).

Overall, LAB fermentation shifted AAM isoflavones from glycoside-rich to aglycone-dominant profiles (Fig. 1 and Tables S2–S4). Although β-glucosidase activity was not directly measured, the observed conversion pattern is consistent with LAB-associated deglycosylation reported for *Lactobacillus* spp. (Delgado et al., 2019). The peak at 36 h aligns with reports describing maximal conversion within 24–48 h in *L.* *plantarum* and related strains (Letizia et al., 2023; Peng et al., 2018), with subsequent attenuation potentially reflecting reduced enzymatic activity, substrate depletion, and/or further metabolism of liberated aglycones. Among conditions tested, a 1:1 LAB02:BMK484 ratio fermented for 36 h produced the most aglycone-dominant profile. This optimized condition was therefore applied in subsequent experiments comparing isoflavone profiles and bioactivities of dried, pasteurized/sterilized, and fermented AAM to quantitatively assess fermentation benefits relative to physical processing.

### 3.2 Changes in physicochemical properties of AAM by food processing stages

The physicochemical properties of AAM significantly varied across food processing stages (Supplementary Table S5). The pH progressively decreased from 6.32 in DrAAM to 4.47 in FeAAM, whereas the titratable acidity increased from 0.50% to 1.62%. Similarly, the °Brix (%) values slightly increased throughout the processing stages, with FeAAM showing the highest value at 18.00%. With regard to the viable cell counts, DrAAM showed no viable LAB, whereas FeAAM showed a significantly higher total viable count (10.2 × 10^9^ CFU/g), mainly because of LAB02 (8.35 × 10^9^ CFU/g) and BMK484 (1.67 × 10^9^ CFU/g). These results confirm that optimized fermentation conditions led to marked changes in acidity, sugar content, and microbial viability, distinguishing FeAAM as a functionally enhanced product. The initial moisture content was 44.49% (StAAM), and it remained at 39.62% (FeAAM) after 36 h; the weight loss during fermentation was 4.87%.

Carbohydrate is the major component of AAM. Studies have shown that the AAM powders contain approximately 40% carbohydrate and starch and approximately 20% soluble sugar (Ito & Arai, 2021; Ogasawara et al., 2006). Protein is known to be the second most abundant component (Neacsu et al., 2021). The starch and protein that are present in the cell walls of plants such as AAM can be degraded into smaller molecules upon heat treatment (Cho et al., 2022). This degradation may contribute to the increase in the °Brix value of StAAM. In addition, some reducing sugars and basic amino acids may undergo nonenzymatic browning reactions upon heating, leading to the production of organic acids, which may contribute to the observed decrease in the pH (Huang et al., 2025). Furthermore, consistent with previous findings, the growth of LAB increased during fermentation, resulting in the production of lactic acid. Accordingly, FeAAM also showed a decrease in pH and an increase in acidity (Cho et al., 2022).

### 3.3 Changes in fatty acid composition of AAM by food processing stages

The fatty acid composition of AAM across processing stages is summarized in Supplementary Table S6. Total saturated fatty acids increased from 125.8 mg/100 g (DrAAM) to 144.2 mg/100 g (FeAAM), with palmitic (C16:0) and stearic acids (C18:0) as the major components. However, the absolute level of saturated fatty acids in FeAAM was only 144.2 mg/100 g (less than 1%), suggesting that the difference primarily reflects a minor compositional shift rather than a nutritionally meaningful increase. Among unsaturated fatty acids, linoleic acid (C18:2c) was the most abundant in all groups, followed by oleic acid (C18:1c) and α-linolenic acid (C18:3n3). Oleic and α-linolenic acid contents were significantly higher in FeAAM than in DrAAM (*p* < 0.05), indicating that fermentation was associated with measurable changes in the unsaturated fatty-acid profile under the present conditions, while the major unsaturated fatty acids remained predominant across processing stages.

**Fig. 2:**
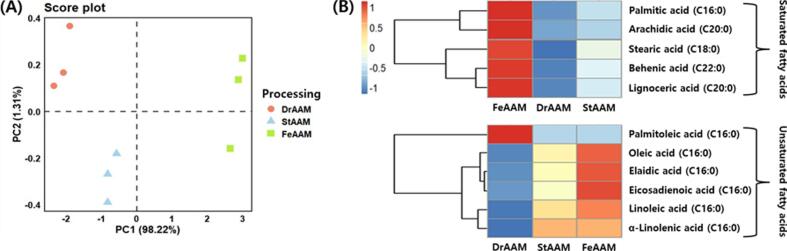
Comparison of fatty acids in *Apios americana* Medikus (AAM) during the food processing stages. (A) Score plot of principal component analysis and (B) correlation heatmap analysis. Food processing stages: DrAAM, dried AAM; StAAM, sterilized AAM; and FeAAM, fermented AAM. AMM was fermented using a 1:1 mixture of *L. plantarum* LAB02 and *L. brevis* BMK484 at 30 °C for 36 h. SFA, saturated fatty acids; USFA, unsaturated fatty acids. The values of various conditions were normalized and clustered in the heatmap. The color displays the intensity of the normalized mean values of different parameters. Statistical significance was considered at *p* < 0.05.

The PCA results based on fatty acid profiles are presented in Fig. 2A. PC1 accounted for 98.22% of the variance and distinctly clustered the samples into DrAAM, StAAM, and FeAAM groups. The heatmap visualized the relative distribution of individual fatty acids, highlighting an enrichment of saturated fatty acids in FeAAM and a greater abundance of unsaturated fatty acids in DrAAM and StAAM (Fig. 2B). Together, these results suggest that fermentation influences the lipid profile of AAM, particularly by increasing saturated fatty acids while maintaining key bioactive unsaturated fatty acids, including oleic and α-linolenic acids.

Several pathways can induce lipid rancidity, including thermal hydrolysis, oxidation, microbial activity, and enzymatic reactions (Wang et al., 2023). In particular, hydrolysis caused by heat and moisture or catalyzed by esterase and phospholipase generates free fatty acids as the ester bonds between glycerol and fatty acids are cleaved (Bornscheuer, 2002). During sterilization, the high-temperature treatment may promote the thermal hydrolysis of lipids that are present in AAM, thereby leading to the formation of a small amount of free fatty acids. However, considering that AAM has a relatively low lipid content, the change in the free fatty acid content during sterilization and fermentation is considered negligible (Neacsu et al., 2021). The fermentation of soybean leaves with a mixed culture of L. *plantarum* and L. *brevis* resulted in notable increases in palmitic, stearic, and oleic acids and a decrease in behenic acid and other fatty acids (Lee, Lee, et al., 2024). Conversely, soybean fermentation with L. *casei* resulted in reductions in palmitic and stearic acids (Li et al., 2020). Collectively, these findings, together with the results of the present study, indicate that lactic acid fermentation of legumes does not follow a consistent trend in fatty acid modulation, likely reflecting substrate-dependent and strain-specific lipase activities.

### 3.4 Changes in free amino acid composition of AAM by food processing stages

**Fig. 3:**
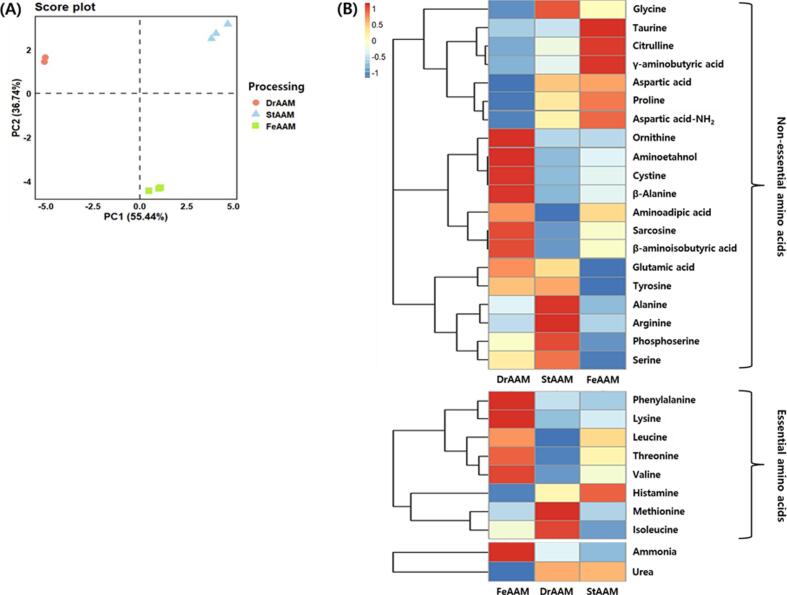
Comparison of free amino acids in *Apios americana* Medikus (AAM) during the food processing stages. (A) Score plot of principal component analysis and (B) correlation heatmap analysis. Food processing stages: DrAAM, dried AAM; StAAM, sterilized AAM; and FeAAM, fermented AAM. AMM was fermented using a 1:1 mixture of *L. plantarum* LAB02 and *L. brevis* BMK484 at 30 °C for 36 h. EAA, essential amino acids; NEAA, nonessential amino acids. The values of various conditions were normalized and clustered in the heatmap. The color displays the intensity of the normalized mean values of different parameters. Statistical significance was considered at *p* < 0.05.

The analytical results of the FAA content in AAM processed under different conditions are shown in Supplementary Table S7. The total FAA content varied noticeably depending on the processing stage, with the highest concentration being observed in StAAM (716.64 mg/100 g), followed by FeAAM and DrAAM. Glutamic acid, aspartic acid, and proline were the dominant nonessential amino acids, whereas leucine, isoleucine, and threonine were the major essential amino acids found in all groups. The results of PCA based on the FAA profiles are presented in Fig. 3A. A clear separation among DrAAM, StAAM, and FeAAM was observed along PC1 (55.44%) and PC2 (36.74%), indicating processing-dependent alterations in the amino acid composition. The heatmap in Fig. 3B further shows differences in individual amino acids.

DrAAM was characterized by elevated levels of glutamic acid and glycine, which contribute to umami and sweetness, whereas FeAAM showed relatively higher GABA and aspartic acid–NH₂ concentrations, suggesting enhanced functional attributes through fermentation. Several LAB species (e.g., *L.* *brevis* and *L. plantarum*) express glutamate decarboxylase (GAD), and LAB fermentation commonly increases GABA through GAD-mediated decarboxylation of L-glutamate (Tram et al., 2025). Consistent with this mechanism, co-fermentation of AAM with *L.* *plantarum* LAB02 and *L.* *brevis* BMK484 decreased glutamic acid while increasing GABA, supporting glutamate-to-GABA conversion during fermentation. Previous studies have reported GABA-enriched legume- and cereal-based products produced using high GABA–producing LAB strains, including a soybean-based GABA-fortified beverage (6.92–6.98 mg/L; Montagano et al., 2025) and GABA-enriched brown rice (8.46–25.09 mg/100 g; Tram et al., 2025). In comparison, FeAAM exhibited a greater magnitude of GABA enhancement, indicating a strong potential for GABA enrichment. In addition to GABA accumulation, LAB co-fermentation increased aspartic acid content. This contrasts with fungal fermentation of AAM, which showed strain-dependent amino acid changes (Lee, Kim, et al., 2024), and aligns with reports on LAB-fermented soy products (Gan et al., 2023). Aspartic acid is metabolically linked to glutamate biosynthesis and broader amino acid metabolism (Chen et al., 2005). Collectively, these results indicate that LAB fermentation remodels the amino acid profile of AAM, including enhanced GABA accumulation. However, as this study assessed compositional changes only, potential functional implications should be interpreted with caution and require confirmation in future bioactivity or in vivo studies.

### 3.5 Changes in mineral composition of AAM by food processing stages

Mineral contents of AAM processed by drying, sterilization (steaming), and LAB fermentation are summarized in Supplementary Table S8. Total mineral levels were comparable across stages, with FeAAM showing a modestly higher value (18.03 mg/100 g) than DrAAM (17.20 mg/100 g) and StAAM (16.87 mg/100 g). Potassium (K), sulfur (S), phosphorus (P), and calcium (Ca) were the predominant minerals in all samples, and FeAAM exhibited slightly higher P and Ca than the other groups. Notably, copper (Cu) and iron (Fe) were detected only in FeAAM, suggesting that fermentation may facilitate the release of trace minerals under the applied analytical conditions.

PCA based on mineral composition (Supplementary Fig. S5A) separated samples by processing stage along PC1 (37.82%) and PC2 (29.72%), indicating stage-dependent mineral profiles. The correlation heatmap (Supplementary Fig. S5B) further highlighted these patterns: FeAAM clustered separately, driven by higher Cu, Fe, and Ca, whereas DrAAM was characterized by relatively higher S and K. Collectively, these results suggest that LAB fermentation is associated with distinct shifts in mineral distribution and increased extractability of selected minerals in AAM.

In this study, the analytically extractable levels of P, K, Ca, and Mg increased after fermentation. One plausible mechanism is phytate degradation. Phytic acid is a major storage form of phosphorus in plant tissues and can be hydrolyzed by phytases to release inorganic phosphate and inositol (Gupta et al., 2015). Although fungi and some bacteria are established phytase producers, phytase activity has also been reported in lactic acid bacteria, including *Lactobacillus* spp. (Sharma et al., 2020; Yang et al., 2022). In support, Arjmand et al. (2023) reported reduced phytic acid in quinoa fermented with *L.* *casei* and *L.* *plantarum*, suggesting that LAB fermentation of AAM may similarly reduce phytate and increase the extractable P fraction. Because phytate forms complexes with divalent minerals, its hydrolysis may also increase extractable Ca and Mg by weakening phytate–mineral interactions (Gupta et al., 2015). In parallel, fermentation-derived organic acids lower pH and can modify mineral binding to proteins and other macromolecules, potentially promoting mineral release and increasing measured extractability (Çabuk et al., 2018). However, phytate content/phytase activity and mineral bioaccessibility were not directly assessed in this study; thus, these mechanistic explanations remain hypothesis-driven. Importantly, the increases reported here reflect analytically extractable mineral levels under the applied extraction and detection conditions and should not be interpreted as improved bioavailability, which requires confirmation using in vitro bioaccessibility assays (e.g., simulated gastrointestinal digestion) and/or absorption-relevant models.

### 3.6 Changes in isoflavone composition of AAM by food processing stages

**Fig. 4:**
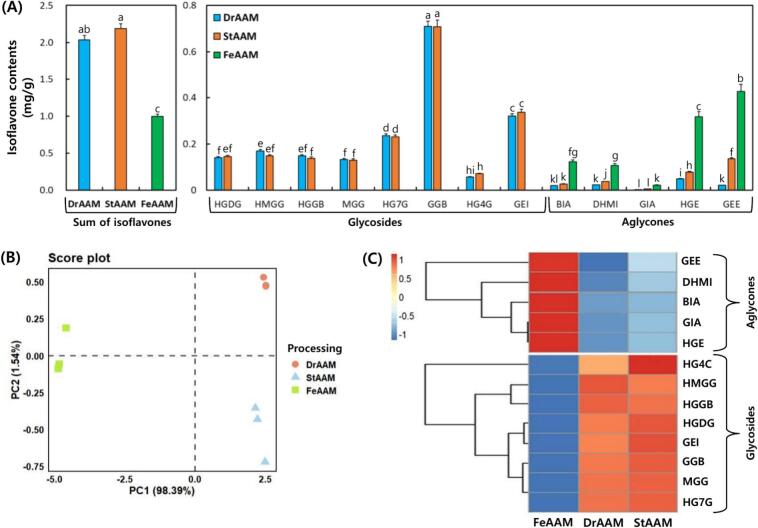
Comparison of isoflavones in *Apios americana* Medikus (AAM) during the food processing stages. (A) Isoflavone contents, (B) score plot of principal component analysis, and (C) correlation heatmap analysis of isoflavones. Food processing stages: DrAAM, dried AAM; StAAM, sterilized AAM; and FeAAM, fermented AAM. AMM was fermented using a 1:1 mixture of *L. plantarum* LAB02 and *L. brevis* BMK484 at 30 °C for 36 h. Isoflavones: HGDG, 2′-hydroxygenistein-4′,7-*O*-diglucoside; HMGG, 2′-hydroxy, 5-methoxygenistein-7-*O*-glucoside; HGGB, 2′-hydroxygenistein-7-*O*-gentibioside; MGG, 5-methoxygenistein-7-*O*-gucoside; HG7G, 2′-hydroxygenistein-7-*O*-glucoside; GGB, genistein-7-*O*-gentibioside; HG4G, 2′-hydroxygenistein-4′-*O*-glucoside; GEI, genistin; BIA, barpisoflavone A; DHMI, 4′,7-dihydroxy-5-methoxyisoflavone; GIA, gerontoisoflavone; HGE, 2′-hydroxygenistein; and GEE, genistein. Different small letters on the bar correspond to significant differences, as determined by Tukey's multiple comparison test (*p* < 0.05). The values of various conditions were normalized and clustered in the heatmap. The color displays the intensity of the normalized mean values of different parameters. Statistical significance was considered at *p* < 0.05.

**Fig. 5:**
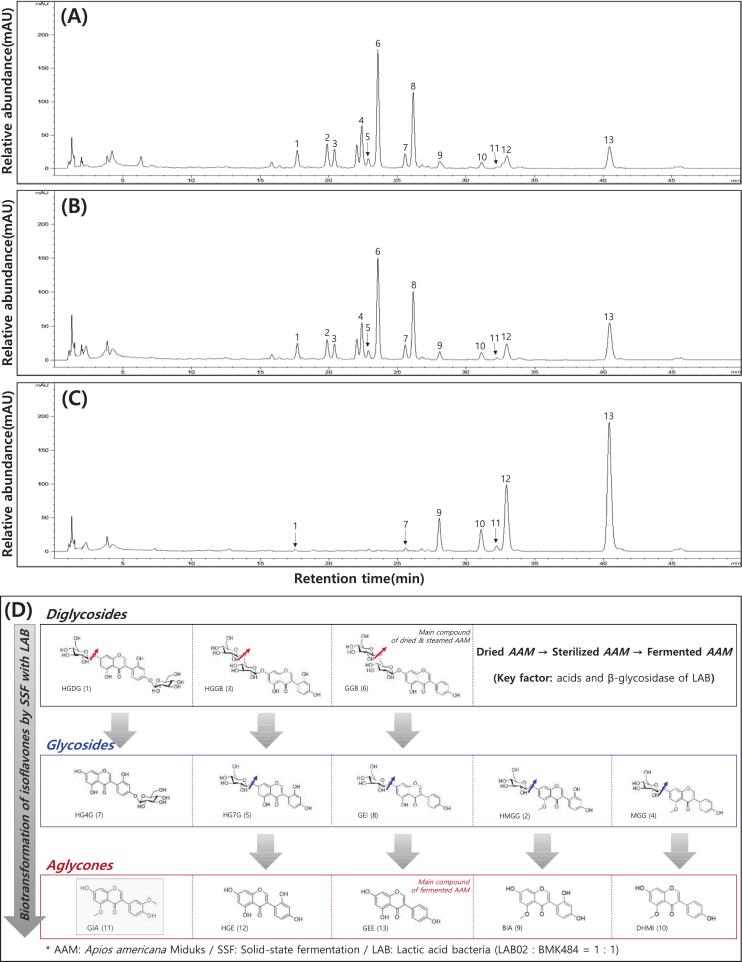
Typical UPLC chromatogram of isoflavones and bioconversion mechanism of isoflavone compounds of *Apios americana* Medikus (AAM) during the food processing stages. (A) DrAAM, dried AAM, (B) StAAM, sterilized AAM; (C) FeAAM, fermented AAM. AMM was fermented using a 1:1 mixture of *L. plantarum* LAB02 and *L. brevis* BMK484 at 30 °C for 36 h; and (D) This schematic summarizes the stepwise biotransformation of AAM isoflavones during processing (Dried → Sterilized → Fermented AAM) and co-fermentation with LAB (SSF; *L. plantarum* LAB02 + *L. brevis* BMK484, 1:1). Isoflavones: HGDG (1), 2′-hydroxygenistein-4′,7-O-diglucoside; HMGG, 2′-hydroxy, 5-methoxygenistein-7-O-glucoside (2); HGGB, 2′-hydroxygenistein-7-O-gentibioside (3); MGG, 5-methoxygenistein-7-O-gucoside (4); HG7G, 2′-hydroxygenistein-7-O-glucoside (5); GGB, genistein-7-O-gentibioside (6); HG4G, 2′-hydroxygenistein-4′-O-glucoside (7); GEI, genistin (8); BIA, barpisoflavone A (9); DHMI, 4′,7-dihydroxy-5-methoxyisoflavone (10); GIA, gerontoisoflavone (11); HGE, 2′-hydroxygenistein (12); and GEE, genistein (13).

Isoflavone profiles of AAM subjected to drying, sterilization (steaming), and LAB fermentation are shown in Fig. 4, Fig. 5. StAAM exhibited the highest total isoflavone content (2.188 mg/g), followed by DrAAM (2.033 mg/g), whereas FeAAM showed a lower total content (0.996 mg/g). Glycoside-type isoflavones (notably GGB and HG7G) predominated in DrAAM and StAAM. In contrast, FeAAM showed a marked decrease in glycosides accompanied by a significant increase in aglycones, including GEE (0.427 mg/g) and HGE (0.317 mg/g) (Fig. 4A). This compositional shift is consistent with fermentation-driven deglycosylation, plausibly mediated by microbial β-glucosidase related activity.

PCA based on isoflavone composition (Fig. 4B) clearly separated samples by processing stage along PC1 (98.39%) and PC2 (1.54%), indicating distinct stage-dependent variation in isoflavone profiles. The heatmap (Fig. 4C) further highlighted these differences, with FeAAM clustering separately due to higher aglycone levels, while DrAAM and StAAM clustered together with relatively higher glycoside abundance. Representative UPLC chromatograms (Fig. 5A–C) supported these trends, showing attenuation/disappearance of glycoside peaks and the emergence of aglycone peaks in FeAAM. Fig. 5D summarizes the proposed bioconversion scheme across processing stages and during LAB fermentation: grey downward arrows indicate a progressive shift from more highly conjugated forms (diglycosides/glycosides) to less conjugated forms (aglycones), and red/blue arrows denote putative sugar-cleavage sites. Compounds labeled as the “main compound of dried & sterilized AAM” or “main compound of fermented AAM” represent the predominant isoflavone forms at each stage as identified and quantified by UPLC. The pathway also illustrates stage-dependent transitions of key glycosides (GGB and HG7G) toward corresponding aglycones (GEE, HGE, and BIA) during LAB fermentation, consistent with the observed increase in deglycosylated forms. However, enzyme activities and bioavailability were not directly evaluated in this study.

Consistent with previous reports, co-fermentation with *L.* *plantarum* and *L.* *brevis* can remodel isoflavone composition toward aglycone enrichment (Lee et al., 2019), and AAM showed a similar trend after fermentation. Isoflavone-containing materials are known to undergo processing-dependent changes, with fermentation often inducing larger compositional shifts than heat treatment alone (Lee et al., 2025). In line with this, AAM exhibited greater conversion to aglycone forms during fermentation than during sterilization (Lee et al., 2025). Evidence on AAM fermentation remains limited; in fungal fermentation, strains with stronger fermentative capacity produced the largest increase in GEE, followed by HGE (Lee, Kim, et al., 2024). FeAAM showed a comparable pattern (Lee et al., 2025; Seo et al., 2018), indicating that AAM glycosides can be converted to aglycones not only by fungal fermentation but also by LAB co-fermentation. Collectively, these results indicate that fermentation promotes deglycosylation of AAM isoflavones and enriches aglycone forms, which are generally considered more functionally relevant than glycosides, although in vivo bioavailability was not assessed here.

### 3.7 Changes in phenolic profiles and biofunctional properties of AAM by food processing stages

The changes in biological activity of AAM across different processing steps are presented in Table 1. The TPC increased in FeAAM (0.68 mg GAE/g) compared with DrAAM (0.57 mg GAE/g) and StAAM (0.59 mg GAE/g), indicating that fermentation enhanced the pool of Folin–Ciocalteu–reactive, redox-active compounds. In contrast, the TFC followed a different pattern, peaking in StAAM (0.96 mg RE/g) relative to DrAAM (0.83 mg RE/g), but decreasing after fermentation (0.76 mg RE/g). The increase in TFC after sterilization may be attributed to enhanced extractability resulting from heat-induced disruption of the plant matrix, which facilitates the release of bound flavonoids. In contrast, the reduced TFC observed in FeAAM is more likely explained by microbial biotransformation of flavonoids into structurally modified derivatives that exhibit lower reactivity in aluminum chloride–based colorimetric assays, rather than a true depletion of total phenolic compounds (Nicolescu et al., 2025; Pękal & Pyrzynska, 2014).

**Table 1:** Total total phenolic and flavonoid contents and antioxidant and digestive enzyme inhibitory activities in *Apios americana* Medikus at different food processing stages (dry, steam, and fermentation) using optimal conditions with LAB02 and BMK484 strains.

**Contents**^a^	**Food processing stages**^b^		
	**DrAAM**	**StAAM**	**FeAAM**
Total phenolic contents (GAE mg/g)	0.57 ± 0.01b	0.59 ± 0.00b	0.68 ± 0.02a
Total flavonoid contents (RE mg/g)	0.83 ± 0.03b	0.96 ± 0.02a	0.76 ± 0.02c
Ferric reducing/antioxidant power (FeSO_4_7H_2_O mg/g)	1.26 ± 0.05c	1.33 ± 0.06b	1.43 ± 0.07a
DPPH radical scavenging IC_50_ (mg/mL)	94.48 ± 3.77a	86.71 ± 4.28b	78.72 ± 3.12c
ABTS radical scavenging IC_50_ (mg/mL)	14.01 ± 0.56a	13.61 ± 0.61ab	11.06 ± 0.55b
Glucosidase inhibition IC_50_ (mg/mL)	123.29 ± 6.15a	113.51 ± 4.52b	102.45 ± 5.12c
Pancreatic-lipase inhibition IC_50_ (mg/mL)	145.77 ± 7.22a	136.88 ± 6.12b	124.18 ± 5.98c

a All values are presented as the *mean ±* SD of triplicate determination. Different letters correspond to the significant differences relating to samples using Duncan's multiple range test (*p* < 0.05).

b Food processing stages: DrAAM, dried *Apios americana* Medikus; StAAM, sterilized *Apios americana* Medikus; FeAAM, fermented *Apios americana* Medikus. AAM was fermented at 30 °C for 36 h using the cockatiel *L.plantarum* LAB02 and *L. brevis* BMK484 (1:1).

Antioxidant capacity increased progressively across processing stages, as indicated by higher FRAP values (1.26 to 1.43 mg FeSO₄·7H₂O/g) and lower DPPH IC₅₀ values (94.48 to 78.72 mg/mL). ABTS scavenging also improved in FeAAM (IC₅₀, 11.06 mg/mL) relative to DrAAM (14.01 mg/mL). In parallel, fermentation enhanced enzyme-inhibitory activity, decreasing IC₅₀ values for α-glucosidase (123.29 to 102.45 mg/mL) and pancreatic lipase (145.77 to 124.18 mg/mL). Notably, these functional gains occurred despite the lower TFC in FeAAM, suggesting that bioactivity is driven more by qualitative remodeling of phenolic composition than by total flavonoid abundance (Pękal & Pyrzynska, 2014; Sytar et al., 2018). This pattern is consistent with fermentation-mediated isoflavone remodeling, in which glycoside-type isoflavones are deglycosylated to aglycones via microbial β-glucosidase related activities (Delgado et al., 2019). Aglycones typically exhibit stronger reducing and radical-scavenging capacity than glycosides, likely due to greater hydroxyl accessibility and improved electron donation (Hwang et al., 2021; Chitisankul & Shimada, 2022). They have also been reported to interact more favorably with digestive enzymes, supporting enhanced inhibition of carbohydrate- and lipid-digesting enzymes (Zhao, Hu, Lu & Zhang, 2022). Accordingly, the elevated TPC together with improved antioxidant and enzyme-inhibitory activities, despite reduced TFC, can be attributed to a shift from glycoside-rich phenolic pools toward aglycone-enriched and/or otherwise biotransformed profiles with higher functional efficacy. Overall, LAB fermentation appeared more effective than thermal processing alone in upgrading AAM bioactivity through targeted phenolic bioconversion, including isoflavone deglycosylation (Hur et al., 2014).

## 4 Conclusions

In this study, LAB co-fermentation of AAM was systematically optimized by screening strain combinations and then refining the inoculum ratio and fermentation time using *L.* *plantarum* LAB02 and *L.* *brevis* BMK484. The selected condition (1:1, 36 h) was associated with efficient isoflavone remodeling, characterized by an increased proportion of aglycone-type isoflavones (including GEE and HGE), together with elevated GABA and aspartic acid levels and enhanced antioxidant capacity (DPPH and ABTS). Fermentation also accompanied measurable changes in the fatty acid profile and analytically measured mineral extractability/solubility (P, K, Ca, and Mg) under the applied analytical conditions, relative to non-fermented processing stages. Overall, these results indicate that LAB co-fermentation is an effective processing strategy to modulate the chemical composition of AAM and to enrich selected bioactive constituents in a time-dependent manner. However, to establish physiological relevance, further studies are warranted focusing on: in vitro bioaccessibility and systemic exposure (e.g., simulated gastrointestinal digestion and plasma/urinary metabolite profiling), metabolic outcomes related to glucose and lipid regulation, and systemic oxidative stress/inflammation using established biomarkers. In particular, the observed increases in analytically extractable/soluble minerals should be further evaluated using bioaccessibility- and absorption-oriented approaches to distinguish analytical extractability from nutritional utilization. Given the elevated GABA content, any implications for neurophysiological outcomes should be considered exploratory and evaluated in dedicated models.

## CRediT authorship contribution statement

**Hee Yul Lee:** Writing – review & editing, Writing – original draft, Validation, Investigation. **Hyo Seon Kim:** Resources, Methodology, Formal analysis. **Ga Young Lee:** Writing – original draft, Visualization, Resources, Formal analysis. **Young Hye Seo:** Investigation, Data curation. **Du Yong Cho:** Validation, Software, Methodology. **Jong Bin Jeong:** Software, Formal analysis. **Mu Yeun Jang:** Visualization, Data curation. **Da Hyun Kim:** Formal analysis, Data curation. **Do Yun Bang:** Formal analysis, Data curation. **Hye Rim Kim:** Formal analysis, Data curation. **Ye Rim Jeong:** Formal analysis, Data curation. **Jun Lee:** Writing – review & editing, Investigation, Funding acquisition, Conceptualization. **Kye Man Cho:** Writing – review & editing, Supervision, Project administration, Funding acquisition.

## Funding

This work was supported by the Basic Science Research Program through the National Research Foundation (NRF) funded by the Ministry of Education (Grant number RS-2023-00245096) and by the project “Development of Innovative Technologies for the Future Value of Herbal Medicine Resources (KSN2511030)” from the Korea Institute of Oriental Medicine (KIOM), Republic of Korea.

## Declaration of competing interest

The authors declare that they have no known competing financial interests or personal relationships that could have appeared to influence the work reported in this paper.

## Data Availability

Data will be made available on request.

## Appendix A Supplementary data

Supplementary material 1: The supplemental materials include: 1) The calibration curve of 13 isoflavone standards (Supplementary Table S1); 2) Comparison of isoflavone contents in the fermented AAM using single and mixed starters with LAB strains (Supplementary Table S2); 3) Comparison of isoflavone contents in fermented AAM with various mixing ratios of LAB strains (Supplementary Table S3); 4) Comparison of isoflavone contents in fermented AAM with different fermentation times (Supplementary Table S4); 5) Comparison of the physicochemical properties, viable cell number, and moisture content in AAM at different food processing stages (dry, steam, and fermentation) using optimal conditions with strains LAB02 and BMK484 (Supplementary Table S5); 6) Comparison of fatty acid contents in AAM at different food processing stages (dry, steam, and fermentation) using optimal conditions with strains LAB02 and BMK484 (Supplementary Table S6); 7) Comparison of free amino acid contents in AAM at different food processing stages (dry, steam, and fermentation) using optimal conditions with strains LAB02 and BMK484 (Supplementary Table S7); 8) Comparison of mineral contents in AAM at different food processing stages (dry, steam, and fermentation) using optimal conditions with strains LAB02 and BMK484 (Supplementary Table S8). Image 1

Supplementary material 2: The supplemental materials include: 1) Typical UPLC chromatograms of 13 isoflavone derivatives and chemical structures (Supplementary Fig. S1); 2) Lactic acid fermentation process of AAM by the cockatiel L. plantarum LAB02 and L. brevis BMK484 (Supplementary Fig. S2); 3) Nucleotide sequence of 16S rRNA from L. brevis LAB02 (Supplementary Fig. S3); 4) Phylogenetic placement by 16S rRNA sequences of L. brevis strain LAB02(Supplementary Fig. S4); 5) Comparison of minerals in AAM during food processing stages (Supplementary Fig. S5). Image 2
