# Supplementary material for: Enhancement of isoflavone aglycones, GABA, and mineral bioavailability in Apios americana Medikus by co-fermentation with Lactiplantibacillus plantarum LAB02 and Levilactobacillus brevis BMK484
Source: Food Chem X. 2026 Mar 10;35:103745. doi: 10.1016/j.fochx.2026.103745 (PMC13053864; doi:10.1016/j.fochx.2026.103745)
Supplement: Supplementary file 1 — Supplementary material 1: The supplemental materials include: 1) The calibration curve of 13 isoflavone standards (Supplementary Table S1); 2) Comparison of isoflavone contents in the fermented AAM using single and mixed starters with LAB strains (Supplementary Table S2); 3) Comparison of isoflavone contents in fermented AAM with various mixing ratios of LAB strains (Supplementary Table S3); 4) Comparison of isoflavone contents in fermented AAM with different fermentation times (Supplementary Table S4); 5) Comparison of the physicochemical properties, viable cell number, and moisture content in AAM at different food processing stages (dry, steam, and fermentation) using optimal conditions with strains LAB02 and BMK484 (Supplementary Table S5); 6) Comparison of fatty acid contents in AAM at different food processing stages (dry, steam, and fermentation) using optimal conditions with strains LAB02 and BMK484 (Supplementary Table S6); 7) Comparison of free amino acid contents in AAM at different food processing stages (dry, steam, and fermentation) using optimal conditions with strains LAB02 and BMK484 (Supplementary Table S7); 8) Comparison of mineral contents in AAM at different food processing stages (dry, steam, and fermentation) using optimal conditions with strains LAB02 and BMK484 (Supplementary Table S8). [file mmc1.docx]

**Supplementary materials**

**Table S1.** The calibration curve of 13 isoflavone standards

| **No.** | **Compound name** | **Abbreviation** | **R.T.^a)^ (min)** | **Calibration curve^b)^** | **LOD (µg/mL)^c)^** | **LOQ (µg/mL)^d)^** |
| --- | --- | --- | --- | --- | --- | --- |
| 1 | 2’-hydroxy genistein-4’,7-*O*-diglucoside | HGDG | 11.962 | y = 21125.396 x + 5857.034, R² = 1.000 | 0.0008 | 0.0023 |
| 2 | 2’-hydroxy, 5-methoxy genistein-7-*O*-glucoside | HMGG | 14.614 | y = 27039.467 x + 4370.425, R² = 1.000 | 0.0003 | 0.0010 |
| 3 | 2’-hydroxy genistein-7-*O*-gentibioside | HGGB | 15.339 | y = 22742.355 x + 1965.424, R² = 1.000 | 0.0006 | 0.0017 |
| 4 | 5-methoxy genistein-7-*O-*gucoside | MGG | 16.687 | y = 28802.956 x + 11020.529, R² = 1.000 | 0.0007 | 0.0022 |
| 5 | 2’-hydroxy genistein-7-*O*-glucoside | HG7G | 17.386 | y = 28667.758 x + 17763.078, R² = 0.999 | 0.0003 | 0.0009 |
| 6 | genistein-7-*O*-gentibioside | GGB | 18.622 | y = 28649.542 x + 49381.625, R² = 1.000 | 0.0014 | 0.0042 |
| 7 | 2’-hydroxy genistein-4’-*O*-glucoside | HG4G | 20.455 | y = 33257.739 x + 10150.90, R² = 0.999 | 0.0005 | 0.0015 |
| 8 | genistin | GEI | 21.275 | y = 40308.445x + 31471.200, R² = 1.000 | 0.0008 | 0.0025 |
| 9 | barpisoflavone A | BIA | 23.100 | y = 51484.018 x + 22207.650, R² = 1.000 | 0.0007 | 0.0021 |
| 10 | 4’,7-dihydroxy-5-methoxyisoflavone | DHMI | 25.654 | y = 48646.572 x + 11765.587, R² = 1.000 | 0.0006 | 0.0020 |
| 11 | gerontoisoflavone | GIA | 26.883 | y = 47709.025 x + 10584.697, R² = 0.999 | 0.0008 | 0.0024 |
| 12 | 2’-hydroxygenistein | HGE | 27.137 | y = 45076.004 x + 11954.163, R² = 0.999 | 0.0007 | 0.0023 |
| 13 | genistein | GEE | 32.223 | y = 54147.209 x + 14500.947, R² = 1.000 | 0.0006 | 0.0019 |
| ^a)^R.T.: Retention time.  ^b)^All values are means of determination in three independent experiments.  ^c)^Limit of detection  ^d)^Limit of quantification | | | | |  |  |

**Table S2.** Comparison of isoflavone contents in the fermented AAM using single and mixed starters with LAB strains

| **Contents^a)^**  **(mg/g)** | **Fermentation by different strains^b)^** | | | | | | | |
| --- | --- | --- | --- | --- | --- | --- | --- | --- |
|  | **CTL^c)^** | **LAB02** | **WCP02** | **BMK184** | **BMK484** | **LAB02**  **+ WCP02** | **LAB02**  **+ BMK184** | **LAB02**  **+ BMK484** |
| **Isoflavone glycosides^d)^** | | | | | | | | |
| HGDG | 0.311 ± 0.001a | 0.015 ± 0.000d | 0.047 ± 0.001b | 0.034 ± 0.001c | 0.031 ± 0.001c | nd^e)^ | nd | nd |
| HMGG | 0.246 ± 0.000a | nd | 0.090 ± 0.001b | 0.073 ± 0.000b | 0.076 ± 0.001b | nd | nd | nd |
| HGGB | 0.272 ± 0.001a | nd | 0.057 ± 0.000b | 0.051 ± 0.001b | 0.049 ± 0.000b | nd | nd | nd |
| MGG | 0.233 ± 0.003a | nd | 0.056 ± 0.001b | 0.045 ± 0.000b | 0.051 ± 0.001b | nd | nd | nd |
| HG7G | 0.205 ± 0.008a | nd | 0.160 ± 0.003b | 0.127 ± 0.003b | 0.113 ± 0.001b | nd | nd | nd |
| GGB | 1.617 ± 0.014a | nd | 0.246 ± 0.003b | 0.204 ± 0.001b | 0.161 ± 0.004b | nd | nd | nd |
| HG4G | 0.020 ± 0.000a | nd | 0.099 ± 0.001b | 0.079 ± 0.003b | 0.074 ± 0.000b | nd | nd | nd |
| GEI | 0.181 ± 0.002a | nd | 0.150 ± 0.002b | 0.080 ± 0.001b | 0.071 ± 0.001b | nd | nd | nd |
| Total | 3.085 | 0.015 | 0.815 | 0.693 | 0.626 | nd | nd | nd |
| **Isoflavone aglycones** | | | | | | | | |
| BIA | 0.002 ± 0.000d | 0.142 ± 0.000a | 0.099 ± 0.001c | 0.112 ± 0.001b | 0.118 ± 0.001b | 0.163 ± 0.002a | 0.151 ± 0.000a | 0.161 ± 0.001a |
| DHMI | 0.007 ± 0.000d | 0.127 ± 0.000a | 0.098 ± 0.002b | 0.109 ± 0.000b | 0.112 ± 0.001b | 0.138 ± 0.001a | 0.132 ± 0.001a | 0.137 ± 0.001a |
| GIA | tr^f)^ | 0.029 ± 0.000c | 0.023 ± 0.000c | 0.025 ± 0.000c | 0.026 ± 0.000c | 0.031 ± 0.000b | 0.030 ± 0.000b | 0.030 ± 0.001b |
| HGE | 0.059 ± 0.002d | 0.280 ± 0.002a | 0.150 ± 0.001b | 0.260 ± 0.001b | 0.256 ± 0.002b | 0.373 ± 0.001a | 0.341 ± 0.001a | 0.377 ± 0.001a |
| GEE | 0.028 ± 0.000d | 0.448 ± 0.003a | 0.269 ± 0.002b | 0.524 ± 0.002b | 0.480 ± 0.002b | 0.622 ± 0.002a | 0.584 ± 0.001a | 0.643 ± 0.001a |
| Total | 0.096 | 1.026 | 0.639 | 1.03 | 0.992 | 1.327 | 1.238 | 1.348 |
| **Sum of**  **isoflavones** | 3.181 | 1.041 | 1.454 | 1.723 | 1.618 | 1.327 | 1.238 | 1.348 |
| ^a )^ All values are presented as the mean *±* SD of triplicate determination. Different letters correspond to the significant differences relating to samples using Duncan’s multiple range test (*p* < 0.05).  ^b)^ AAM was fermented for 72 h at 30°C using single and mixed LAB.  ^c)^ CTL (control): not fermentation (0 h).  ^d)^ Isoflavones: HGDG, 2’-hydroxy genistein-4’,7-*O*-diglucoside; HMGG, 2’-hydroxy, 5-methoxy genistein-7-*O*-glucoside; HGGB, 2’-hydroxy genistein-7-*O*-gentibioside; MGG, 5-methoxy genistein-7-*O-*gucoside; HG7G, 2’-hydroxy genistein-7-*O*-glucoside; GGB, genistein-7-*O*-gentibioside; HG4G, 2’-hydroxy genistein-4’-*O*-glucoside; GEI, genistin; BIA, barpisoflavone A; DHMI, 4’,7-dihydroxy-5-methoxyisoflavone; GIA, gerontoisoflavone; HGE, 2’-hydroxygenistein and GEE, genistein.  ^e)^ nd: not detected.  ^f)^ tr: lower than the quantitation limits. | | | | | | | | |

**Table S3.** Comparison of isoflavone contents in fermented AAM with various mixing ratios of LAB strains

| **Contents^a)^ (mg/g)** | **Fermentation by different LAB strains mixed ratios^b)^** | | | | | |
| --- | --- | --- | --- | --- | --- | --- |
|  | **CTL^c)^** | **1:1** | **1:2** | **1:3** | **2:1** | **3:1** |
| **Isoflavone glycosides^d)^** | | | | | | |
| HGDG | 0.225 ± 0.005a | 0.009 ± 0.000d | 0.009 ± 0.000d | 0.005 ± 0.000d | 0.023 ± 0.000c | 0.004 ± 0.000d |
| HMGG | 0.221 ± 0.002a | 0.028 ± 0.000c | 0.027 ± 0.000c | 0.018 ± 0.001b | 0.054 ± 0.000b | 0.009 ± 0.000c |
| HGGB | 0.224 ± 0.001a | 0.003 ± 0.000d | 0.003 ± 0.000d | 0.003 ± 0.000d | 0.011 ± 0.000b | 0.002 ± 0.000d |
| MGG | 0.177 ± 0.003a | 0.017 ± 0.000d | 0.016 ± 0.000d | 0.009 ± 0.000d | 0.036 ± 0.000b | 0.003 ± 0.000d |
| HG7G | 0.251 ± 0.001a | 0.031 ± 0.000c | 0.033 ± 0.000c | 0.013 ± 0.000d | 0.078 ± 0.001b | 0.001 ± 0.000d |
| GGB | 1.112 ± 0.006a | tr^e)^ | tr | tr | tr | tr |
| HG4G | 0.056 ± 0.000c | 0.119 ± 0.001a | 0.107 ± 0.000a | 0.083 ± 0.002b | 0.137 ± 0.001a | 0.026 ± 0.000c |
| GEI | 0.340 ± 0.002a | 0.026 ± 0.000d | 0.037 ± 0.000b | 0.010 ± 0.000d | 0.057 ± 0.003b | tr |
| Total | 2.606 | 0.233 | 0.232 | 0.141 | 0.396 | 0.045 |
| **Isoflavone aglycones** | | | | | | |
| BIA | 0.022 ± 0.001d | 0.154 ± 0.002a | 0.145 ± 0.001b | 0.132 ± 0.002c | 0.120 ± 0.001c | 0.151 ± 0.002a |
| DHMI | 0.028 ± 0.001d | 0.142 ± 0.001a | 0.132 ± 0.001b | 0.123 ± 0.002c | 0.116 ± 0.000c | 0.130 ± 0.000b |
| GIA | 0.003 ± 0.000d | 0.032 ± 0.000a | 0.030 ± 0.000b | 0.027 ± 0.000c | 0.027 ± 0.000c | 0.030 ± 0.000b |
| HGE | 0.078 ± 0.001d | 0.403 ± 0.003a | 0.329 ± 0.001b | 0.300 ± 0.003c | 0.269 ± 0.005c | 0.345 ± 0.001b |
| GEE | 0.091 ± 0.001d | 0.811 ± 0.016a | 0.566 ± 0.012b | 0.493 ± 0.005c | 0.509 ± 0.013b | 0.510 ± 0.001a |
| Total | 0.222 | 1.542 | 1.202 | 1.075 | 1.041 | 1.166 |
| **Sum of isoflavones** | 2.828 | 1.775 | 1.434 | 1.216 | 1.437 | 1.211 |
| ^a )^ All values are presented as the *mean ±* SD of triplicate determination. Different letters correspond to the significant differences relating to samples using Duncan’s multiple range test (*p* < 0.05).  ^b)^ AAM was fermented for 72h at 30°C using a mixture of various ratios (1:1, 1:2, 1:3, 2:1, and 3:1) of *L. plantarum* LAB02 and *L. brevis* BMK484.  ^c)^ CTL (control): not fermentation (0 h).  ^d)^ Isoflavones: HGDG, 2’-hydroxy genistein-4’,7-*O*-diglucoside; HMGG, 2’-hydroxy, 5-methoxy genistein-7-*O*-glucoside; HGGB, 2’-hydroxy genistein-7-*O*-gentibioside; MGG, 5-methoxy genistein-7-*O-*gucoside; HG7G, 2’-hydroxy genistein-7-*O*-glucoside; GGB, genistein-7-*O*-gentibioside; HG4G, 2’-hydroxy genistein-4’-*O*-glucoside; GEI, genistin; BIA, barpisoflavone A; DHMI, 4’,7-dihydroxy-5-methoxyisoflavone; GIA, gerontoisoflavone; HGE, 2’-hydroxygenistein and GEE, genistein.  ^e)^ tr: lower than the quantitation limits. | | | | | | |

**Table S4.** Comparison of isoflavone contents in fermented AAM with different fermentation times

| **Contents^a)^ (mg/g)** | **Fermentation times (h)^b)^** | | | | | | |
| --- | --- | --- | --- | --- | --- | --- | --- |
|  | **0** | **12** | **24** | **36** | **48** | **60** | **72** |
| **Isoflavone glycosides^c)^** | | | | | | | |
| HGDG | 0.156 ± 0.005a | 0.062 ± 0.001d | 0.155 ± 0.002a | 0.006 ± 0.001d | 0.116 ± 0.003b | 0.088 ± 0.004b | 0.099 ± 0.002b |
| HMGG | 0.149 ± 0.002a | 0.007 ± 0.000d | 0.098 ± 0.001b | nd^d)^ | tr^e)^ | tr | tr |
| HGGB | 0.171 ± 0.001a | nd | 0.058 ± 0.001b | nd | nd | nd | nd |
| MGG | 0.119 ± 0.001a | tr | 0.079 ± 0.001b | nd | nd | nd | nd |
| HG7G | 0.186 ± 0.001a | tr | 0.055 ± 0.000b | nd | nd | nd | nd |
| GGB | 0.852 ± 0.001a | tr | 0.199 ± 0.002b | nd | nd | nd | nd |
| HG4G | 0.035 ± 0.001c | 0.004 ± 0.000d | 0.017 ± 0.000b | nd | nd | nd | nd |
| GEI | 0.169 ± 0.004a | tr | 0.036 ± 0.000b | nd | nd | nd | nd |
| Total | 1.837 | 0.073 | 0.697 | 0.006 | 0.116 | 0.088 | 0.099 |
| **Isoflavone aglycones** | | | | | | | |
| BIA | 0.031 ± 0.000c | 0.083 ± 0.002a | 0.049 ± 0.000b | 0.114 ± 0.001a | 0.088 ± 0.001b | 0.080 ± 0.002b | 0.080 ± 0.002b |
| DHMI | 0.035 ± 0.000c | 0.084 ± 0.002a | 0.059 ± 0.000b | 0.098 ± 0.001a | 0.098 ± 0.001a | 0.089 ± 0.002a | 0.091 ± 0.002a |
| GIA | 0.006 ± 0.000c | 0.020 ± 0.000a | 0.012 ± 0.000b | 0.022 ± 0.000a | 0.023 ± 0.000a | 0.020 ± 0.000a | 0.021 ± 0.000a |
| HGE | 0.014 ± 0.000d | 0.025 ± 0.000a | 0.030 ± 0.000b | 0.184 ± 0.000a | 0.111 ± 0.000b | 0.052 ± 0.000c | 0.079 ± 0.001b |
| GEE | 0.011 ± 0.000d | 0.013 ± 0.000a | 0.019 ± 0.000b | 0.194 ± 0.001a | 0.118 ± 0.001b | 0.033 ± 0.000d | 0.072 ± 0.001b |
| Total | 0.097 | 0.225 | 0.169 | 0.604 | 0.438 | 0.274 | 0.252 |
| **Sum of isoflavones** | 1.934 | 0.298 | 0.866 | 0.61 | 0.554 | 0.362 | 0.351 |
| ^a)^ All values are presented as the *mean ±* SD of triplicate determination. Different letters correspond to the significant differences relating to samples using Duncan’s multiple range test (*p* < 0.05).  ^b)^ AAM was co-fermented at 30°C with *L. plantarum* LAB02 and *L. brevis* BMK484 (1:1) for up to 72 h, with samples collected at 0, 12, 24, 36, 48, 60, and 72 h.  ^c)^ Isoflavones: HGDG, 2’-hydroxy genistein-4’,7-*O*-diglucoside; HMGG, 2’-hydroxy, 5-methoxy genistein-7-*O*-glucoside; HGGB, 2’-hydroxy genistein-7-*O*-gentibioside; MGG, 5-methoxy genistein-7-*O-*gucoside; HG7G, 2’-hydroxy genistein-7-*O*-glucoside; GGB, genistein-7-*O*-gentibioside; HG4G, 2’-hydroxy genistein-4’-*O*-glucoside; GEI, genistin; BIA, barpisoflavone A; DHMI, 4’,7-dihydroxy-5-methoxyisoflavone; GIA, gerontoisoflavone; HGE, 2’-hydroxygenistein and GEE, genistein.  ^d)^ nd: not detected.  ^e)^ tr: lower than the quantitation limits. | | | | | | | |

**Table S5.** Comparison of the physicochemical properties, viable cell number, and moisture content in AAM at different food processing stages (dry, steam, and fermentation) using optimal conditions with strains LAB02 and BMK484

| **Contents ^a)^** | **Food processing stages ^b)^** | | |
| --- | --- | --- | --- |
|  | **DrAAM** | **StAAM** | **FeAAM** |
| **Physicochemical properties** | | | |
| pH | 6.32 ± 0.16a | 5.97 ± 0.14b | 4.47 ± 0.15c |
| Acidity (%, as lactic acid) | 0.50 ± 0.03b | 0.54 ± 0.03b | 1.62 ± 0.06a |
| Brix (%) | 15.60 ± 0.70b | 17.60 ± 0.65a | 18.00 ± 0.80a |
| **Viable cell numbers** (×10^9^ CFU/g) | | | |
| LAB02 | nd^c)^ | 0.08 ± 0.00b | 8.35 ± 0.26a |
| BMK484 | nd | 0.14 ± 0.00b | 1.67 ± 0.05a |
| Total | nd | 0.22 ± 0.01b | 10.2 ± 0.31a |
| **Moisture content (%)** | - | 44.49 ± 3.30a | 39.62 ± 2.67b |
| ^a)^ All values are presented as the *mean ±* SD of triplicate determination. Different letters correspond to the significant differences relating to samples using Duncan’s multiple range test (*p* < 0.05).  ^b)^ Food processing stages: DrAAM, dried *Apios americana* Medikus; StAAM, sterilized *Apios americana* Medikus; FeAAM, fermented *Apios americana* Medikus. AAM was fermented at 30°C for 36 h using the cockatiel *L. plantarum* LAB02 and *L. brevis* BMK484 (1:1).  ^c)^ nd: not detected. | | | |

**Table S6.** Comparison of fatty acid contents in AAM at different food processing stages (dry, steam, and fermentation) using optimal conditions with strains LAB02 and BMK484

| **Contents^a)^ (mg/100 g)** | **Food processing stages^b)^** | | |
| --- | --- | --- | --- |
|  | **DrAAM** | **StAAM** | **FeAAM** |
| **Saturated fatty acids** |  |  |  |
| Palmitic acid (C16:0) | 92.4 ± 1.85c | 93.4 ± 2.24b | 102.0 ± 2.14a |
| Stearic acid (C18:0) | 19.1 ± 0.46c | 20.8 ± 0.42b | 24.0 ± 0.62a |
| Arachidic acid (C20:0) | 4.4 ± 0.11c | 4.5 ± 0.13b | 4.9 ± 0.10a |
| Behenic acid (C22:0) | 6.0 ± 0.13c | 6.7 ± 0.18b | 8.6 ± 0.21a |
| Lignoceric acid (C24:0) | 3.9 ± 0.10c | 4.1 ± 0.11b | 4.7 ± 0.11a |
| Total | 125.8 | 129.5 | 144.2 |
| **Unsaturated fatty acids** | | | |
| Palmitoleic acid (C16:1) | 1.8 ± 0.04b | nd^c)^ | nd |
| Elaidic acid (C18:1t) | 2.3 ± 0.07c | 2.6 ± 0.07b | 2.9 ± 0.08a |
| Oleic acid (C18:1c) | 29.1 ± 0.79b | 29.6 ± 0.68b | 30.1 ± 0.81a |
| Linoleic acid (C18:2c) | 167.7 ± 3.86b | 176.0 ± 4.40a | 176.3 ± 4.76a |
| α-Linolenic acid (C18:3n3) | 18.7 ± 0.47b | 19.7 ± 0.39a | 19.2 ± 0.44a |
| Eicosadienoic acid (C20:2) | 2.3 ± 0.05c | 2.5 ± 0.06b | 2.8 ± 0.06a |
| Total | 221.9 | 230.4 | 231.3 |
| **Sum of fatty acids** | 347.7 | 359.9 | 375.5 |
| ^a)^ All values are presented as the *mean ±* SD of triplicate determination. Different letters correspond to the significant differences relating to samples using Duncan’s multiple range test (*p* < 0.05).  ^b)^ Food processing stages: DrAAM, dried *Apios americana* Medikus; StAAM, sterilized *Apios americana* Medikus; FeAAM, fermented *Apios americana* Medikus. AAM was fermented at 30°C for 36 h using the cockatiel *L. plantarum* LAB02 and *L. brevis* BMK484 (1:1).  ^c)^ nd: not detected. | | | |

**Table S7.** Comparison of free amino acid contents in AAM at different food processing stages (dry, steam, and fermentation) using optimal conditions with strains LAB02 and BMK484

| **Contents^1)^ (mg/100 g)** | **Food processing stages^b)^** | | |
| --- | --- | --- | --- |
|  | **DrAAM** | **StAAM** | **FeAAM** |
| **Non-essential amino acids** | | | |
| Phosphoserine | 23.72 ± 0.72b | 23.58 ± 0.65b | 21.01 ± 0.61c |
| Taurine | 5.80 ± 0.21b | 5.56 ± 0.18b | 6.01 ± 0.25a |
| Proline | 34.42 ± 0.90b | 40.90 ± 1.10a | 42.99 ± 1.20a |
| Aspartic acid | 87.51 ± 2.51b | 106.09 ± 3.09a | 105.55 ± 3.05a |
| Serine | 16.34 ± 0.55b | 20.23 ± 0.71a | 5.67 ± 0.18c |
| Aspartic acid - NH_2_ | 136.47 ± 4.47b | 163.27 ± 4.81a | 179.73 ± 5.10a |
| Glutamic acid | 54.07 ± 1.12a | 47.97 ± 1.10b | 33.12 ± 1.20c |
| Sarcosine | 12.11 ± 0.35a | 10.23 ± 0.27b | 10.49 ± 0.32b |
| Aminoadipic acid | 3.70 ± 0.12a | 2.53 ± 0.09b | 3.25 ± 0.10a |
| Glycine | 20.26 ± 0.86c | 39.46 ± 1.34a | 28.98 ± 1.15b |
| Alanine | 28.90 ± 1.19b | 34.19 ± 1.40a | 24.74 ± 0.92c |
| Citrulline | 1.47 ± 0.08c | 3.22 ± 0.12b | 6.82 ± 0.20a |
| Cystine | 8.05 ± 0.32a | 5.96 ± 0.24b | 6.17 ± 0.28a |
| Tyrosine | 6.76 ± 0.26a | 6.59 ± 0.22a | 3.54 ± 0.14c |
| β-alanine | 15.49 ± 0.52a | 11.42 ± 0.35b | 11.86 ± 0.45b |
| β-aminoisobutyric acid | 9.69 ± 0.31a | 6.53 ± 0.22b | 7.52 ± 0.26b |
| γ-aminobutyric acid | 49.10 ± 1.50c | 53.94 ± 1.60b | 72.76 ± 2.10a |
| Aminoetahnol | 6.40 ± 0.23a | 3.13 ± 0.12b | 3.64 ± 0.16b |
| Ornithine | 6.11 ± 0.21b | 4.00 ± 0.18c | 3.90 ± 0.15c |
| Arginine | 22.69 ± 0.82b | 23.99 ± 0.88a | 20.82 ± 0.77c |
| Total | 549.06 | 612.79 | 598.57 |
| **Essential amino acids** | | | |
| Threonine | 14.63 ± 0.60b | 16.77 ± 0.72a | 15.17 ± 0.50a |
| Valine | 9.66 ± 0.36b | 11.00 ± 0.42a | 9.66 ± 0.34b |
| Methionine | 4.58 ± 0.17a | 2.66 ± 0.10b | 2.55 ± 0.09b |
| Isoleucine | 24.38 ± 1.06a | 12.69 ± 0.55b | 5.52 ± 0.18c |
| Leucine | 13.86 ± 0.55b | 21.17 ± 0.85a | 19.20 ± 0.72a |
| Phenylalanine | 11.45 ± 0.42b | 13.78 ± 0.46a | 10.35 ± 0.38c |
| Lysine | 10.85 ± 0.38b | 15.78 ± 0.55a | 10.92 ± 0.36b |
| Histamine | 14.26 ± 0.50b | 10.02 ± 0.30c | 15.63 ± 0.52a |
| Total | 103.67 | 103.87 | 89 |
| **Sum of free amino acids** | 652.73 | 716.64 | 687.56 |
| Ammonia | 50.46 ± 1.40b | 53.15 ± 1.50a | 44.31 ± 1.25c |
| Urea | 353.31 ± 10.00a | 271.87 ± 9.50c | 314.39 ± 9.00b |
| ^a)^ All values are presented as the *mean ±* SD of triplicate determination. Different letters correspond to the significant differences relating to samples using Duncan’s multiple range test (*p* < 0.05).  ^b)^ Food processing stages: DrAAM, dried *Apios americana* Medikus; StAAM, sterilized *Apios americana* Medikus; FeAAM, fermented *Apios americana* Medikus. AAM was fermented at 30°C for 36 h using the cockatiel *L. plantarum* LAB02 and *L. brevis* BMK484 (1:1). | | | |

**Table S8.** Comparison of mineral contents in AAM at different food processing stages (dry, steam, and fermentation) using optimal conditions with strains LAB02 and BMK484

| **Contents^a)^ (mg/100 g)** | **Food processing stages^b)^** | | |
| --- | --- | --- | --- |
|  | **DrAAM** | **StAAM** | **FeAAM** |
| Phosphorus (P) | 2.08 ± 0.04c | 2.13 ± 0.06b | 2.23 ± 0.05a |
| Sulfur (S) | 1.03 ± 0.03b | 0.57 ± 0.01c | 1.07 ± 0.02a |
| Potassium (K) | 12.22 ± 0.33b | 12.22 ± 0.29b | 12.89 ± 0.37a |
| Calcium (Ca) | 0.62 ± 0.02b | 0.61 ± 0.01b | 0.66 ± 0.02a |
| Copper (Cu) | nd^c)^ | nd | 0.01 ± 0.00a |
| Iron (Fe) | 0.04 ± 0.00a | 0.04 ± 0.00a | 0.04 ± 0.00a |
| Magnesium (Mg) | 0.56 ± 0.01b | 0.55 ± 0.02b | 0.59 ± 0.02a |
| Manganese (Mn) | 0.01 ± 0.00a | 0.01 ± 0.00a | 0.01 ± 0.00a |
| Zinc (Zn) | 0.02 ± 0.00a | 0.02 ± 0.00a | 0.02 ± 0.00a |
| Aluminium (Al) | 0.05 ± 0.00a | 0.05 ± 0.00a | 0.05 ± 0.00a |
| Boron (B) | 0.05 ± 0.00a | 0.05 ± 0.00a | 0.04 ± 0.00b |
| Natrium (Na) | 0.27 ± 0.01b | 0.39 ± 0.01a | 0.36 ± 0.01a |
| Silicon (Si) | 0.25 ± 0.01b | 0.23 ± 0.01b | 0.23 ± 0.01b |
| **Sum of minerals** | **17.20** | **16.87** | **18.20** |
| ^a)^ All values are presented as the *mean ±* SD of triplicate determination. Different letters correspond to the significant differences relating to samples using Duncan’s multiple range test (*p* < 0.05).  ^b)^ Food processing stages: DrAAM, dried *Apios americana* Medikus; StAAM, sterilized *Apios americana* Medikus; FeAAM, fermented *Apios americana* Medikus. AAM was fermented at 30°C for 36 h using the cockatiel *L. plantarum* LAB02 and *L. brevis* BMK484 (1:1).  ^c)^ nd: not detected. | | | |
