# Supplementary material for: Enhancement of isoflavone aglycones, GABA, and mineral bioavailability in Apios americana Medikus by co-fermentation with Lactiplantibacillus plantarum LAB02 and Levilactobacillus brevis BMK484
Source: Food Chem X. 2026 Mar 10;35:103745. doi: 10.1016/j.fochx.2026.103745 (PMC13053864; doi:10.1016/j.fochx.2026.103745)
Supplement: Supplementary file 2 — Supplementary material 2: The supplemental materials include: 1) Typical UPLC chromatograms of 13 isoflavone derivatives and chemical structures (Supplementary Fig. S1); 2) Lactic acid fermentation process of AAM by the cockatiel L. plantarum LAB02 and L. brevis BMK484 (Supplementary Fig. S2); 3) Nucleotide sequence of 16S rRNA from L. brevis LAB02 (Supplementary Fig. S3); 4) Phylogenetic placement by 16S rRNA sequences of L. brevis strain LAB02(Supplementary Fig. S4); 5) Comparison of minerals in AAM during food processing stages (Supplementary Fig. S5). [file mmc2.pptx]

## Slide 1
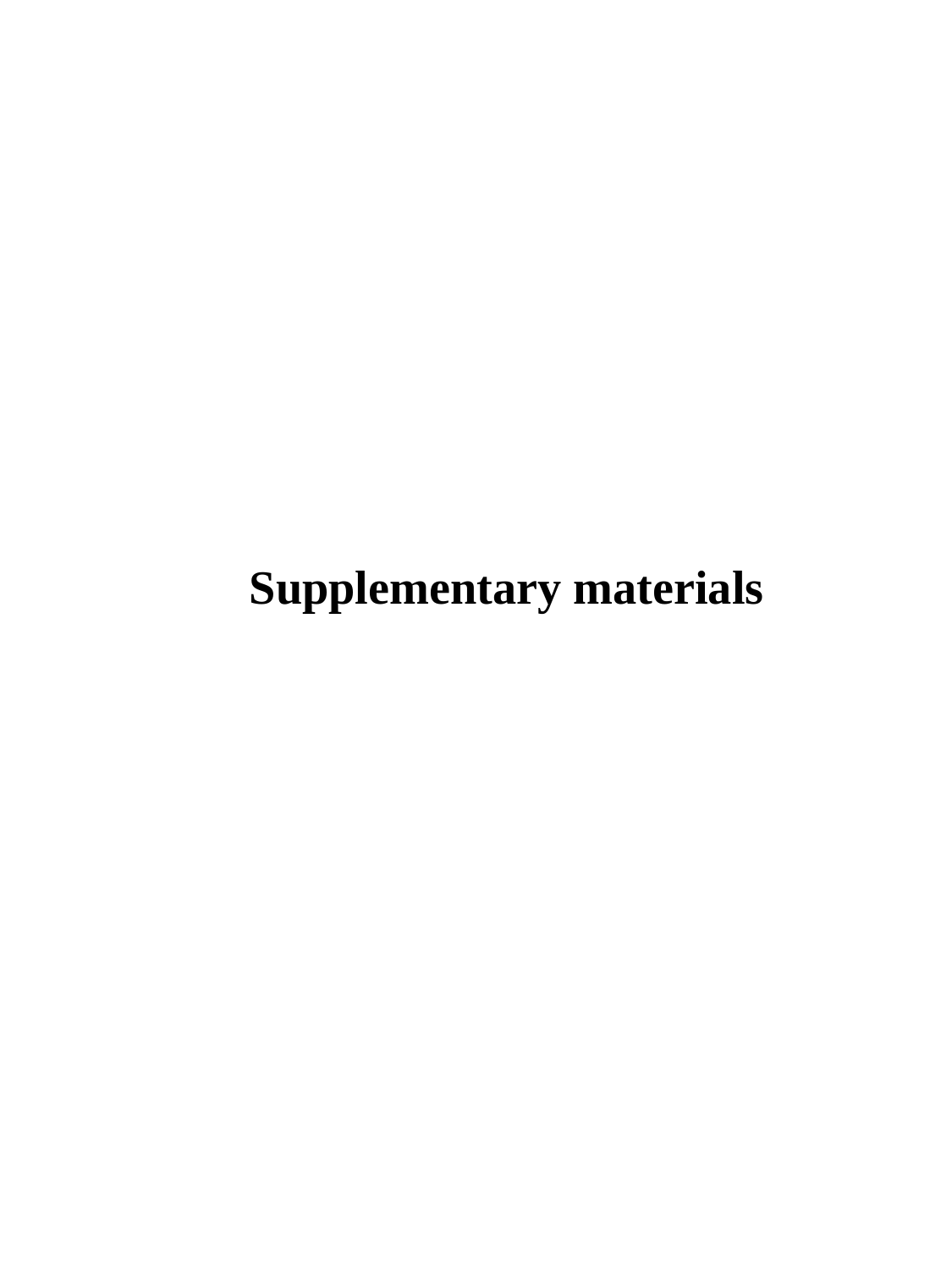

Supplementary materials

## Slide 2
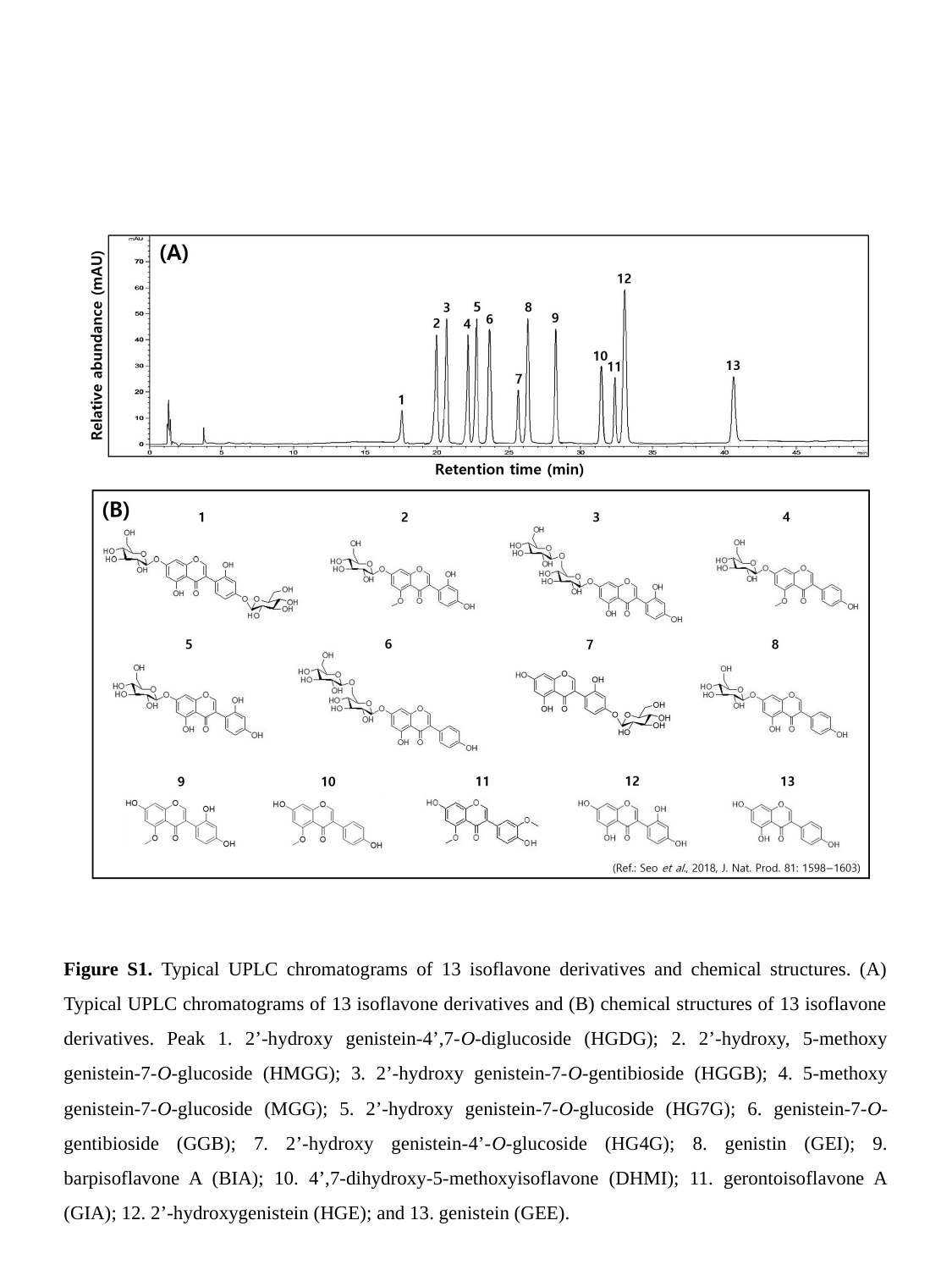

Figure S1. Typical UPLC chromatograms of 13 isoflavone derivatives and chemical structures. (A) Typical UPLC chromatograms of 13 isoflavone derivatives and (B) chemical structures of 13 isoflavone derivatives. Peak 1. 2’-hydroxy genistein-4’,7-O-diglucoside (HGDG); 2. 2’-hydroxy, 5-methoxy genistein-7-O-glucoside (HMGG); 3. 2’-hydroxy genistein-7-O-gentibioside (HGGB); 4. 5-methoxy genistein-7-O-glucoside (MGG); 5. 2’-hydroxy genistein-7-O-glucoside (HG7G); 6. genistein-7-O-gentibioside (GGB); 7. 2’-hydroxy genistein-4’-O-glucoside (HG4G); 8. genistin (GEI); 9. barpisoflavone A (BIA); 10. 4’,7-dihydroxy-5-methoxyisoflavone (DHMI); 11. gerontoisoflavone A (GIA); 12. 2’-hydroxygenistein (HGE); and 13. genistein (GEE).

## Slide 3
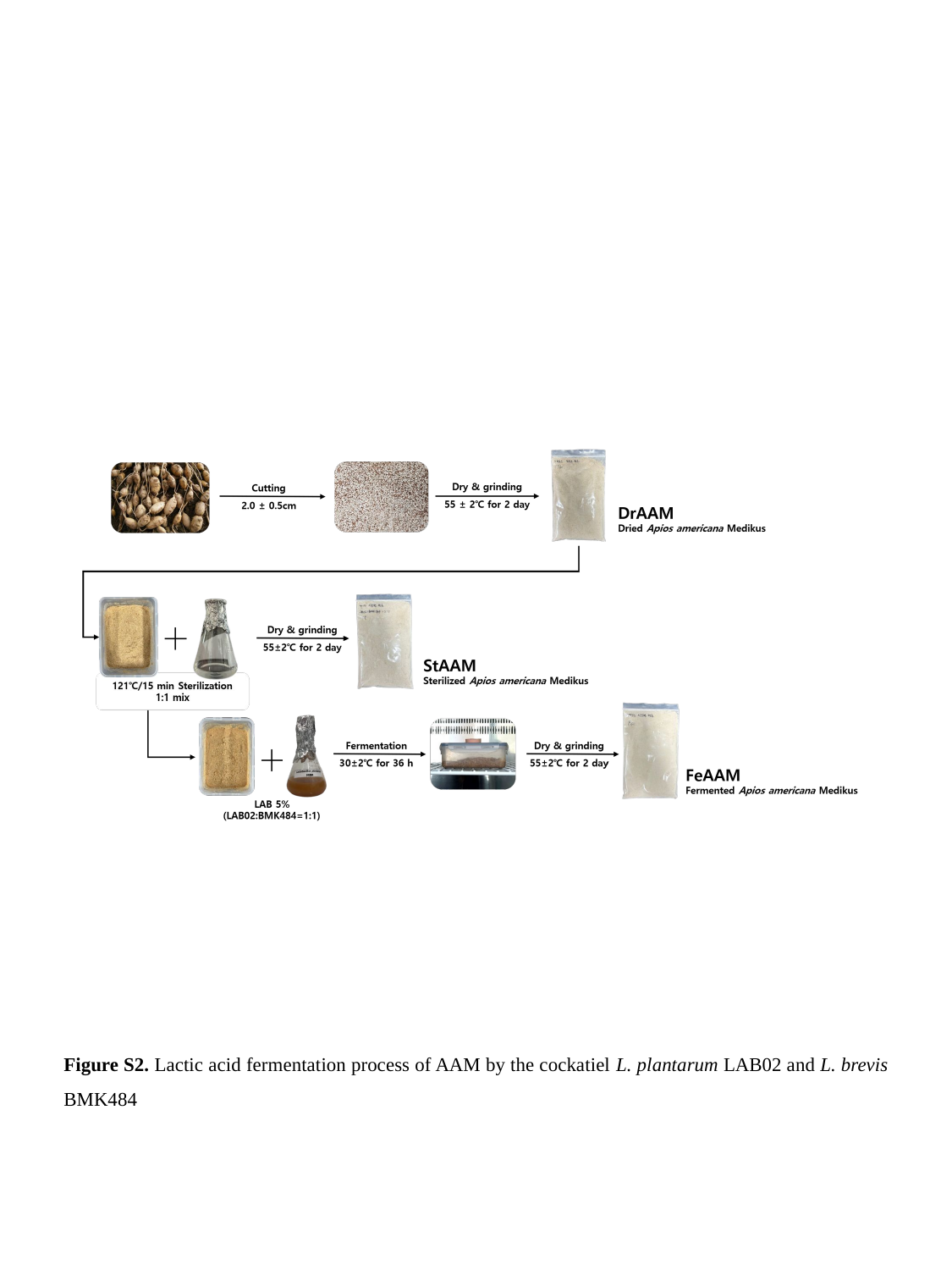

Figure S2. Lactic acid fermentation process of AAM by the cockatiel L. plantarum LAB02 and L. brevis BMK484

## Slide 4
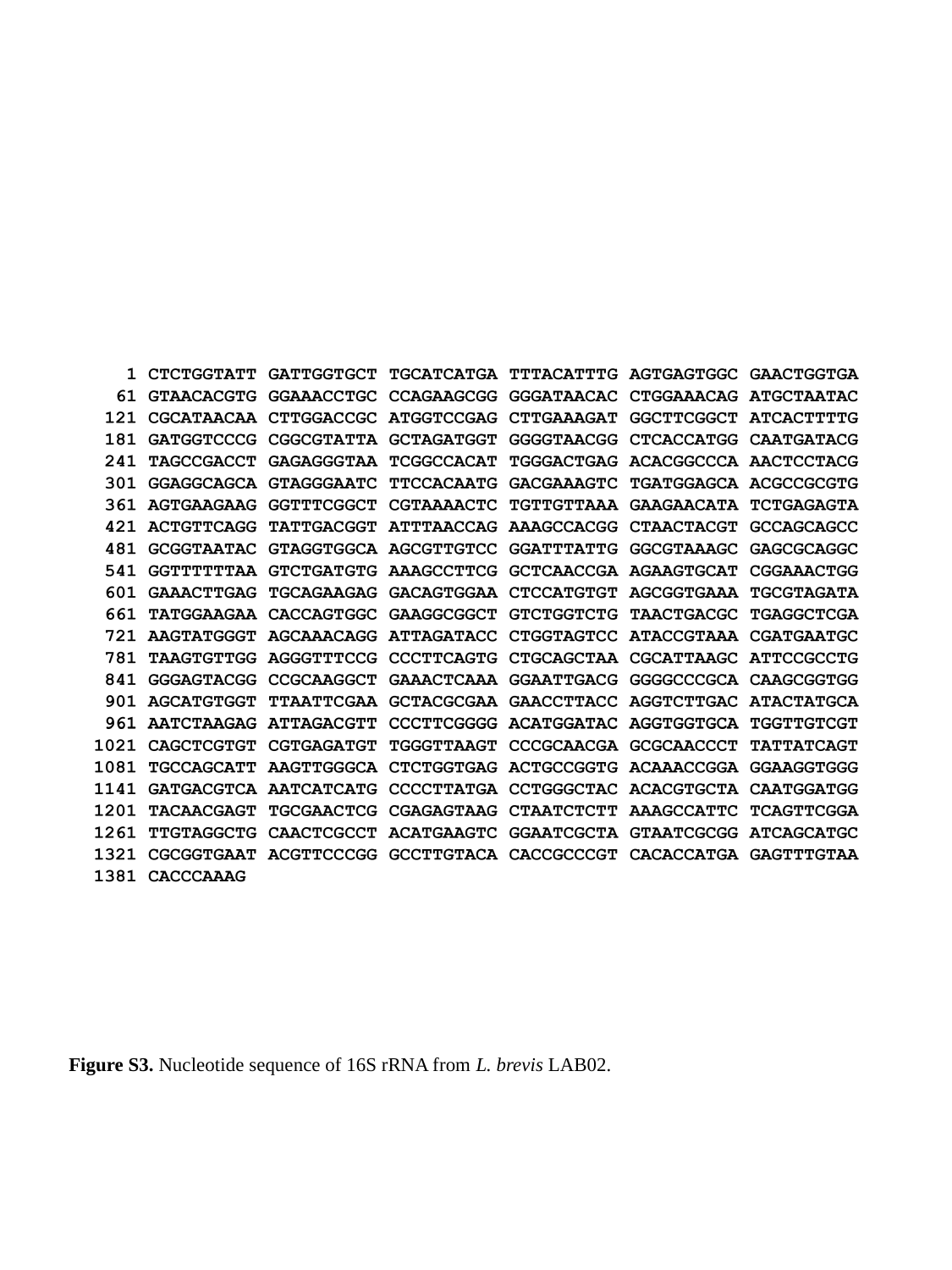

Figure S3. Nucleotide sequence of 16S rRNA from L. brevis LAB02.

## Slide 5
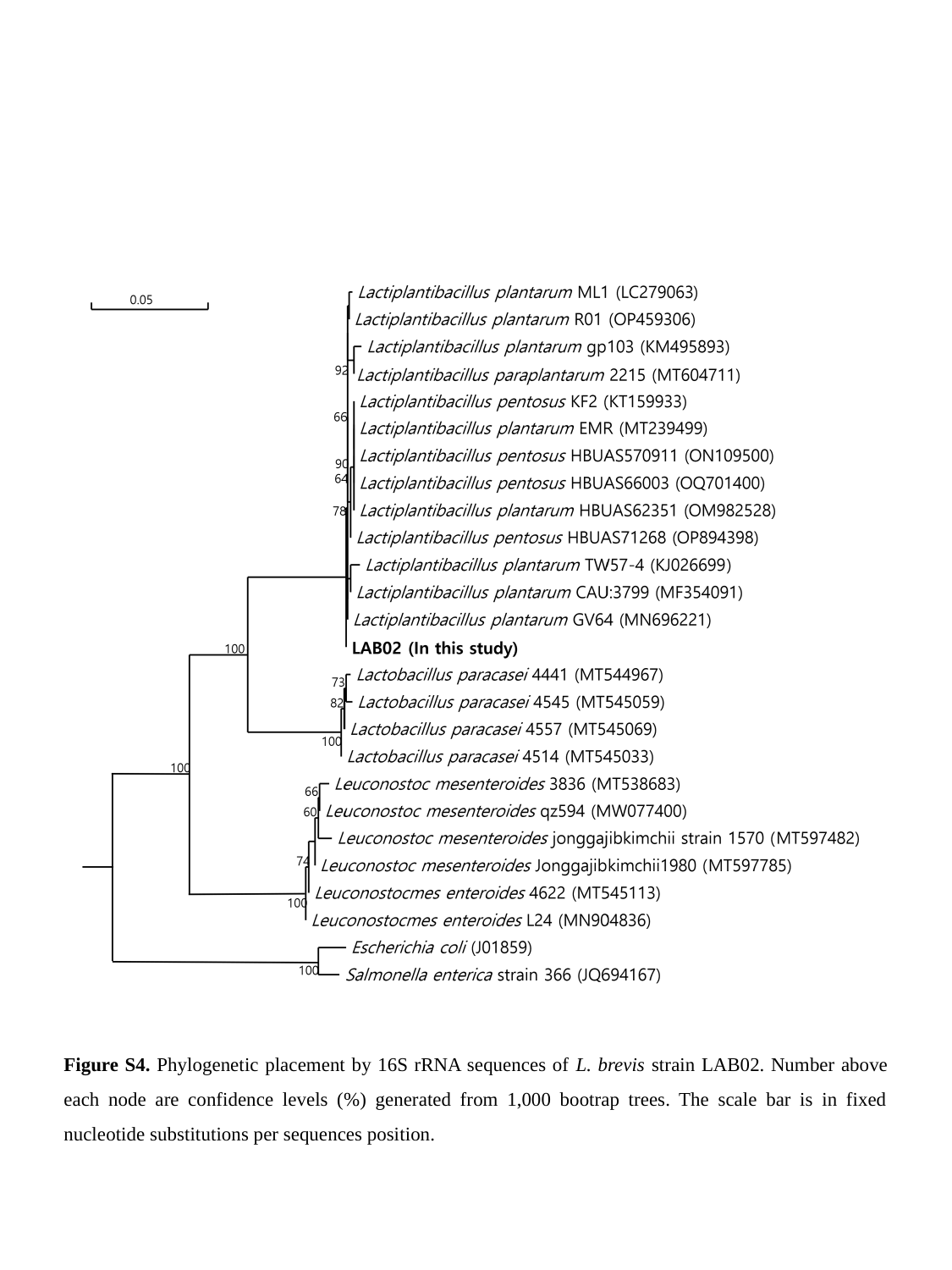

Figure S4. Phylogenetic placement by 16S rRNA sequences of L. brevis strain LAB02. Number above each node are confidence levels (%) generated from 1,000 bootrap trees. The scale bar is in fixed nucleotide substitutions per sequences position.

## Slide 6
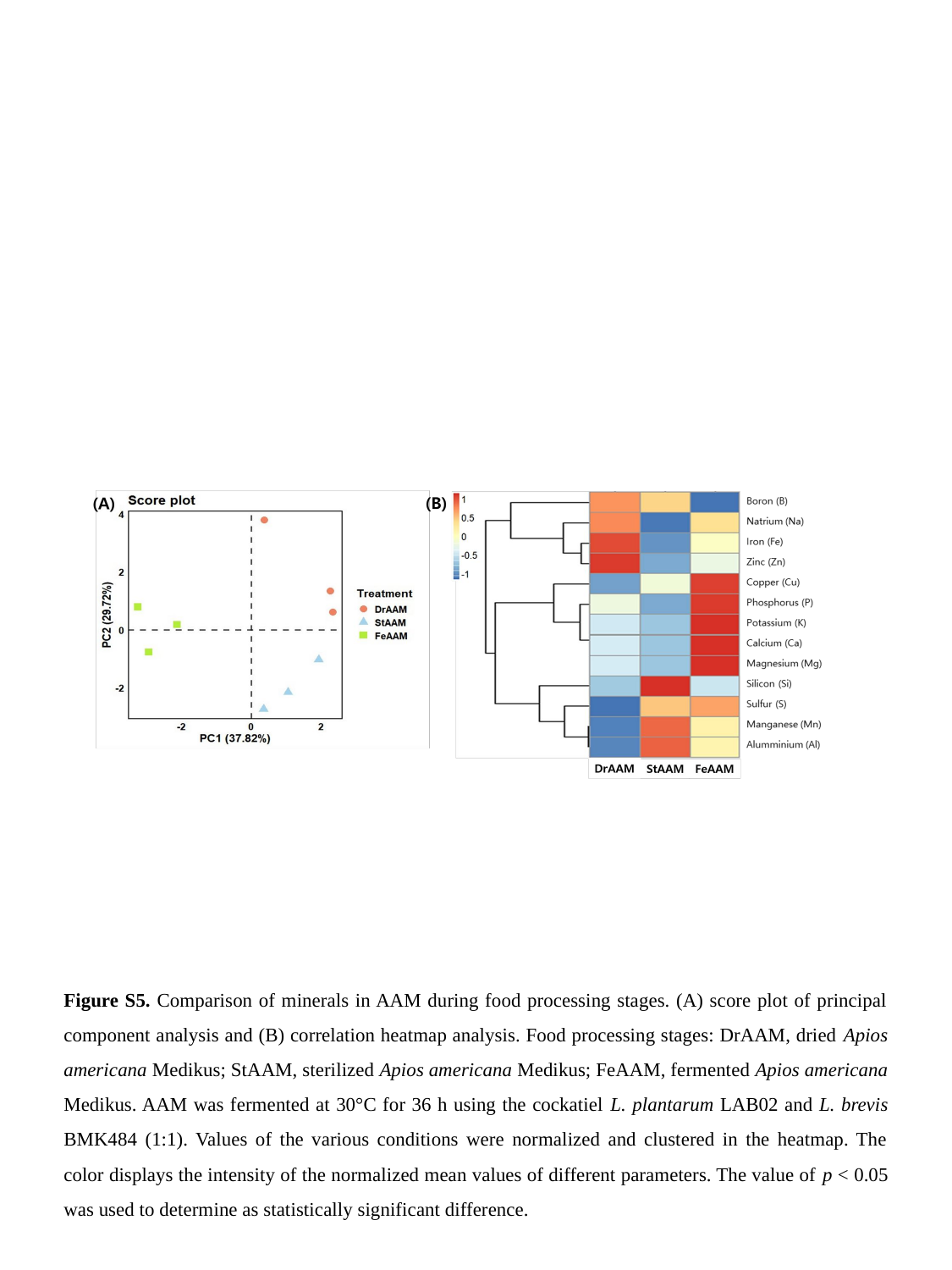

Figure S5. Comparison of minerals in AAM during food processing stages. (A) score plot of principal component analysis and (B) correlation heatmap analysis. Food processing stages: DrAAM, dried Apios americana Medikus; StAAM, sterilized Apios americana Medikus; FeAAM, fermented Apios americana Medikus. AAM was fermented at 30°C for 36 h using the cockatiel L. plantarum LAB02 and L. brevis BMK484 (1:1). Values of the various conditions were normalized and clustered in the heatmap. The color displays the intensity of the normalized mean values of different parameters. The value of p < 0.05 was used to determine as statistically significant difference.
